# Generation of hepatocyte- and endocrine pancreatic-like cells from human induced endodermal progenitor cells

**DOI:** 10.1371/journal.pone.0197046

**Published:** 2018-05-11

**Authors:** Rangarajan Sambathkumar, Renate Akkerman, Sumitava Dastidar, Philip Roelandt, Manoj Kumar, Manmohan Bajaj, Ana Rita Mestre Rosa, Nicky Helsen, Veerle Vanslembrouck, Eric Kalo, Satish Khurana, Jos Laureys, Conny Gysemans, Marijke M. Faas, Paul de Vos, Catherine M. Verfaillie

**Affiliations:** 1 KU Leuven, Interdepartmental Stem Cell Institute, Department of Development and Regeneration, Stem Cell Biology and Embryology, Leuven, Belgium; 2 University of Groningen, University Medical Center Groningen (UMCG), Pathology and Medical Biology, Division of Medical Biology, Section Immunoendocrinology, Groningen, The Netherlands; 3 School of Biology, Indian Institute of Science Education and Research, Thiruvananthapuram, Kerala, India; 4 KU Leuven, Department of Clinical and Experimental Medicine, Clinical and Experimental Endocrinology unit, Leuven, Belgium; 5 University of Groningen, University Medical Center Groningen (UMCG), Department of Obstetrics and Gynecology, Groningen, The Netherlands; University of Kansas Medical Center, UNITED STATES

## Abstract

Multipotent Adult Progenitor Cells (MAPCs) are one potential stem cell source to generate functional hepatocytes or β-cells. However, human MAPCs have less plasticity than pluripotent stem cells (PSCs), as their ability to generate endodermal cells is not robust. Here we studied the role of 14 transcription factors (TFs) in reprogramming MAPCs to induced endodermal progenitor cells (iENDO cells), defined as cells that can be long-term expanded and differentiated to both hepatocyte- and endocrine pancreatic-like cells. We demonstrated that 14 TF-iENDO cells can be expanded for at least 20 passages, differentiate spontaneously to hepatocyte-, endocrine pancreatic-, gut tube-like cells as well as endodermal tumor formation when grafted in immunodeficient mice. Furthermore, iENDO cells can be differentiated *in vitro* into hepatocyte- and endocrine pancreatic-like cells. However, the pluripotency TF *OCT4*, which is not silenced in iENDO cells, may contribute to the incomplete differentiation to mature cells *in vitro* and to endodermal tumor formation *in vivo*. Nevertheless, the studies presented here provide evidence that reprogramming of adult stem cells to an endodermal intermediate progenitor, which can be expanded and differentiate to multiple endodermal cell types, might be a valid alternative for the use of PSCs for creation of endodermal cell types.

## Introduction

In the field of regenerative medicine, the creation of functional mature hepatocytes and insulin producing pancreatic β-cells for cell based therapy of liver diseases and diabetes, respectively, are highly sought after goals. Currently, liver failure is treated by whole liver transplantation or transplantation of primary hepatocytes as a bridge to liver transplantation, while for late stage type 1 diabetes, the whole pancreas or cadaveric islet transplantation is the current treatment of choice[1–3]. However, scarcity of donor organs and immunorejection represent a major hurdle to treat most patients. In addition, the pharmaceutical industry is also in need for reliable drug hepatotoxicity screening models, as drug-induced liver injury is one of the most prevalent causes for drug discovery failure[4]. Although primary human hepatocytes are the best source of cells for liver toxicity assessment, scarcity of donor cells, and rapid dedifferentiation of hepatocytes cultured *in vitro*[5–8] represent major obstacles for these studies.

Human embryonic stem cells (hESCs) and, more recently, induced pluripotent stem cells (iPSCs) are cells that can self-renew long-term and differentiate into cells from the three germ layers[9, 10]. Multiple protocols for *in vitro* differentiation of pluripotent stem cells (PSCs) to hepatocyte like cells (HLCs)[11–15] and mature β-cells[16–18] have been developed by mimicking *in vivo* development. Currently, fully mature hepatocytes cannot yet be created from PSCs[19, 20], while recent studies demonstrated that mature functional β-cells can be derived from PSCs[21, 22].

An alternative to create hepatocytes or β-cells from PSCs is the direct transdifferentiation or lineage conversion of, for instance, fibroblasts into these cell lineages using combinations of transcription factors (TFs) and small molecules. Although cells with hepatocyte-like features can be generated[23–25], they are not fully similar to primary human hepatocytes; by contrast, glucose-stimulated insulin producing β-cells have been generated by direct transdifferentiation[26–33]. One drawback of this approach is that transdifferentiation creates post-mitotic cells that cannot be expanded and that repeated transdifferentiations from fibroblasts, which have limited expansion potential, will be required to create new populations of target cells.

Another alternative would be to generate an expandable pool of intermediate endodermal progenitor cells that can, subsequently be differentiated to mature differentiated endodermal cells. In contrast to PSCs, such endodermal progenitors might represent a safer cell source, as differentiation to non-endodermal cells would not occur. Compared to direct lineage conversion, such endodermal progenitors can be extensively expanded[25, 33].

In this study we choose to use human multipotent adult progenitor cells (hMAPCs) for transdifferentiation to expandable endodermal progenitor cells (termed iENDO cells). The rationale for the use of hMAPCs as starting population was threefold: (1) hMAPCs are derived from human bone marrow and can be expanded significantly (for ±70 Population doublings (PDs)) without acquisition of genetic abnormalities[34]; (2) hMAPCs (trade name MultiStem®) are currently being used clinically in the setting of ischemic disorders and as immunomodulators without known toxicity[35–37] (3) although hMAPCs can differentiate *in vitro* and *in vivo* in mesodermal cell types, including endothelium, they differentiate, unlike their rodent counterparts, less robust to endodermal cell types[14, 34, 38, 39].

We here demonstrated that using a complement of TFs, chosen based on insights from early endoderm fate specification and differentiation, hMAPCs can be reprogrammed to expandable iENDO cells, which could then be differentiated towards hepatocyte- and pancreatic β-cell-like cells *in vitro* and *in vivo*.

## Materials and methods

### Generation of lentiviral vectors carrying transcription factors

We selected 16 candidate TFs: ESC pluripotency factors (*OCT4*, *SOX2*, *KLF4*, *CMYC*), TFs expressed in early definitive endoderm *(MIXL1*, *GATA4*, *SOX17*, *FOXA1*, *FOXA2*, *FOXD3*, *FOXF1)*, TFs expressed in late endoderm *(HNF4α*, *HNF6*, *HNF1α*, *HEX*, *CEBPα)*. Primers were designed with including specific restriction enzyme sites in the flanking regions to amplify the cDNAs (S1 Table). The coding sequence for *FOXA2*, *HNF1α*, *HNF4α*, *CEBPα*, *HNF6*, and *FOXA1*, *FOXF1*, *FOXD3* and for *HEX*, *MIXL1*, *GATA4*, *SOX17* were PCR amplified from hESC differentiated to hepatocytes for 12 days, 6 days and 4 days, respectively and cloned into the PLVX-IRES-HYGRO constitutive CMV promoter based lentiviral vector plasmid purchased from (Cat No. 632185, Clontech, Cambridge, USA). Human *OCT4*, *SOX2*, *KLF4* and *CMYC* cDNA were excised from pMXs-*OCT3/4* (Cat.No. 17217), pMXs-*SOX2* (Cat No. 17218), pMXs-*KLF4* (Cat No. 17219), pMXs-*cMYC* (Cat No. 17220) respectively. The cDNAs were cloned into the PLVX lentiviral vector (Addgene, Cambridge, USA). Each TF construct was verified by colony PCR, restriction digestion pattern and cDNA sequence analysis. Each TF containing lentiviral vector was co-transfected with 2nd generation lentiviral packaging (gag pol tat rev)/envelope (VSV-G) plasmids into Lenti-X™ 293T Cell line purchased (Cat No. 632180, Clontech, CA, USA) using Fugene transfection reagent (Cat No. E2311, Promega, Madison, WI, USA). Transfer vector- 6μg, Packaging plasmid (psPAX2) (Cat No. 12260, Addgene, Cambridge, USA)- 3.5μg, Envelope plasmid (pMD2. G) (Cat No. 12259 Addgene, Cambridge, USA)– 1.5μg. Supernatants containing the lentiviral particles were collected after 48hr, filtered through a 0.45μm filter and stored at -80°C for future use.

### Culture conditions for human MAPCs

Human Multipotent Adult Progenitors (hMAPCs) were derived from human bone marrow as described in Roobrouck et al.[34] MAPCs were cultured as previously described[34]. This cells were cultured in medium containing of 60% DMEM low-glucose (Gibco, Invitrogen, Carlsbad, CA, USA), 40% MCDB-201 (Sigma-Aldrich’s Louis, MO, USA), supplemented with 50mM dexamethasone, 10^−4^ M L-Ascorbic acid, 1x Insulin-Transferrin-Selenium(ITS), 0.5x linoleic acid-bovine serum albumin (LA-BSA)(all from Sigma Aldrich), 1% Penicillin/Streptomycin (Gibco, Invitrogen), along with 2% Serum Supreme (Lonza Biowhittaker, Basel, Switzerland) and human PDGF-BB (R&D Systems, Minneapolis, MN,USA) and human EGF (Sigma-aldrich) (both 10ng/ml). Filter medium through 0.22μm filter (Millipore). Human MAPC-cultures were maintained under hypoxic conditions 37°C and 5% O_2,_ 5% CO_2_ humidified incubator at a density of 400 cells/cm^2^ and were passaged every 2–3 days.

### Generation of induced endodermal progenitors (iENDO) from human MAPCs

On day 0, 45,000 hMAPCs cells were plated in 10 cm^2^ petri dishes (Cat No. 150350, Thermo Scientific™ Nunclon™ Delta treated, VWR International, Belgium) in triplicates. On day 1, cells were transduced with a cocktail of 14 or 16 un-concentrated viral vector supernatants (MOI of 3) with infection efficiency (96.57% at 300μl) (S15A and S15B Fig). On day 4, transduced cells were harvested and replated on 1.8% differentiation Matrigel (Cat No. 356231, BD biosciences, Bedford, MA, USA) coated six well plates (Cat No. 3516, Corning-Costar^®^, MA, USA). A part of the cells was used to evaluate transgene expression. From day 5 onwards, cells were maintained in endoderm induction medium[14] containing 60% DMEM low glucose (Cat No. 31885023, Gibco, Grand Island, NY, USA); 40% MCDB-201 (Cat.No. M6770, Sigma, Saint Louis, MO, USA); 1x-Penicillin-Streptomycin(10,000 U/mL) (Cat No. 15140122, Gibco, Carlsbad, CA, USA); 0.25x LA-BSA (100x) (Cat No. L9530, Sigma, Saint Louis, MO, USA); 0.25x ITS-A (100x) (Cat No. 51300044, Gibco, Grand Island, NY, USA); 100nM,L-ascorbic Acid (Cat No. A4403, Sigma-Aldrich, Saint Louis, MO, USA); 1μM dexamethasone (Cat.No. D2915, Sigma-Aldrich, Saint Louis, MO, USA), 50 μM, 2-mercaptoethanol (50mM) (Cat No. 31350010, Gibco, Grand Island, NY, USA) supplemented with 100ng/ml Activin-A (Cat No. 120-14E), 50ng/ml Wnt3a (Cat No. 315–20) and 5ng/ml BMP4 (Cat No. 120-05ET). All growth factors were purchased from Peprotech, USA. From day 9 to 15 morphological changes from mesenchymal to epithelial cells were assessed by bright field microscopy. Untransduced and PLVX-eGFP empty vector tranduced hMAPCs cultured under similar conditions were used as negative control. The iENDO cells were maintained in a 37°C, 21% O_2_, 5% CO_2_ incubator. Between days 20–25 transcripts for endogenous mesendoderm/definitive endoderm and late endoderm marker genes were measured by qRT-PCR. Cells were fixed with 4% paraformaldehyde (PFA) (Cat No. P6148, Sigma-Aldrich, Saint Louis, MO, USA) overnight at 4°C to perform immunostaining.

### Expansion potential of iENDO cells

14TF iENDO cells were seeded at one million cells/100 cm^2^ petri dish (Cat No. 150350, Thermo Scientific™ Nunclon™ Delta treated, VWR International, Belgium) coated with 0.1% gelatin. Afterwards every 4–5 days, cells were harvested with 0.25% Trypsin EDTA (Cat No. 25200056, Gibco, Grand island, NY, USA) and enumerated using a NUCLEOCOUNTER® NC-100™. Population doublings (PDs) were enumerated as the number of cells initially seeded (C_0_) to the number of cells harvested (C_1_) at each passage using the following equation: PDnew = PD initial + [log (C_1_/C_0_]/log2.

### Maintenance and expansion of hESCs

H9 hESCs (purchased from WiCell, Madison, WI, USA) were expanded on a 6 well plate (Cat No. 3516, Corning-Costar^®^, VWR International, Belgium), in feeder-free conditions on hESC-qualified BD Matrigel^TM^, (Cat No. 354277, BD Biosciences, Bedford, MA, USA) using E8 medium Essential 8^TM^ Basal Medium Essential 8^TM^ Supplement (Cat No. A1517001, Gibco, Grand Island, NY, USA).

### Transduction efficiency in hMAPC

To determine the transduction efficiency of hMAPC, we serially diluted the PLVX-IRES-eGFP lentiviral vector (0–1000μl) and transduced an equal number of hMAPC (50,000 cells/well of 12 well plate). After 3 days, we quantified eGFP expression by FACScanto flow cytometer (BD Biosciences). Data were analyzed with FlowJo Sofware (Tree Star, Ashland, OR, USA).

### iENDO cell transplantation under kidney capsule of immunodeficient BALBC Rag2^-/-^ γc^-/-^ mice

Immunodeficient BALBC Rag2^-/-^ γc^-/-^ mice were maintained in accordance with protocols approved by the ethics committee of KU Leuven. 14 TF iENDO cells were collected from culture dishes by 0.25% trypsin, and injected under the kidney capsule of 10–12-week-old Rag2^-/-^ γc^-/-^ mice (N = 16). After 3 weeks (N = 3) and 3 months (N = 12), mice were sacrificed, the kidneys harvested and washed with 1X PBS 2–5 times. A small part of the graft was collected in lysis buffer for RNA extraction and qRT-PCR analysis, while the remainder was fixed with 4% PFA overnight at 4°C for immunohistochemistry and Hematoxyline and Eosin (H&E) staining. Serum from transplanted and untransplanted mice was collected and stored at -80°C for human albumin and C-peptide analysis.

### Hepatocyte organoid differentiation from 14TF-iENDO cells

14TF iENDO cells were allowed to form 3D organoids by plating 25,000-iENDO cells/well in low attachment 96 well U bottom cell repellent plates (Cat. No.650970, Greiner-bio one, Belgium) by spinning at 300g for 5 min in Liver Differentiation medium (LDM) consisting of 60% DMEM low glucose; 40% MCDB-201; 1x-Penicillin-Streptomycin; (10,000 U/mL)); 0.25x LA-BSA (100x); 0.25x ITS-A (100x); 100nM, L-ascorbic Acid; 1μM dexamethasone; 50μM, 2-mercaptoethanol (50mM) supplemented with in addition as follows, for protocol-A, day 0 to 4: 50 ng/ml aFGF (Cat.No. 100-17A, Peprotech, NJ, USA) with or without 0.6% DMSO (Cat No.D2650 Sigma-Aldrich, Saint Louis, MO, USA) and, subsequently, from day 4 to 20 with 20 ng/ml HGF (Cat No.100-39H, Peprotech, NJ, USA) with or without 2% DMSO. For protocol B, organoids were cultured in 20 ng/ml HGF and 2% DMSO from day 0 to 16. Compacted organoids were maintained in a 37°C, 21% O_2_, 5% CO_2_ incubator, with medium change every 2 days until day 20 and 16 (protocol-A and B respectively). On the last day of differentiation organoids were collected for qRT-PCR. For immunostaining, organoids were fixed with 4% PFA overnight at 4°C and subsequently, stored in 70% ethanol at 4°C until embedding. Differentiation medium was collected and stored at -80°C for albumin ELISA.

### Hepatocyte organoid differentiation from hESCs

Differentiation of hESCs to definitive endoderm and hepatocyte-like cells was performed as described previously by our group[40] with addition of 0.6% DMSO between day 0 and day 12 and 2% DMSO between day 12 and day 28. On day 20, cells were washed with 1x PBS (Cat No. 10010015, Gibco, Grand Island, NY, USA), incubated with collagenase-1 (200U/ml, 300μl for 20 minutes) and detached by pipetting. Cells were collected, spun for 5 minutes at 300g and re-suspended in LDM containing 10% FBS (Cat No. F6178, Sigma-Aldrich, Saint Louis, MO, USA), Revitacell™ (1:100), (Cat No. A2644501, Gibco, Grand Island, NY, USA), 20ng/ml HGF and 2% DMSO. Cells were plated in low attachment round bottom plates at 7000 cells/well in 20μl. After 24hours, compact organoids formed, after which 100μl LDM containing HGF (20ng/ml) and 2% DMSO was added. Subsequently, medium was changed every alternate day. On day 30, medium was collected for albumin ELISA and organoids were harvested for gene expression analysis.

### Pancreatic organoid differentiation from 14TF-iENDO cells

14TF iENDO cells were allowed to form organoids by plating 25,000-iENDO cells/well in low attachment 96-well U-bottom plates by spinning at 300g for 5 min in pancreatic differentiation medium (PDM) consists of 60% DMEM low glucose; 40% MCDB-201; 1x-Penicillin-Streptomycin; 0.25x LA-BSA; 0.5x ITS-A (100x); 100nM L-ascorbic Acid); 50 μM, 2-mercaptoethanol (50mM) without cytokines. Compacted organoids were formed in 2 days. Subsequently organoids were maintained in pancreatic differentiation basal medium supplemented as described below: Stage-1 (day 0–8): PDM with 1% B27® supplement (50x) (Cat No. 17504044, Gibco, Grand Island, NY, USA), 0.1mM Sodium butyrate (Cat No. B5887, Sigma-Aldrich, Saint Louis, MO, USA), 1μM T3 (3,3′,5-triiodo-L-thyronine sodium salt), (Cat No. 64245, Millipore, Calbiochem, San Diego, CA, USA), 3.5mM D-glucose (Cat.No. G7021 Sigma-Aldrich, Saint Louis, MO, USA), 100ng/ml Noggin (Cat No. 120-10C, Peprotech, USA), 5μM ALK5-II Inhibitor (Cat No. ALX-270-445, Enzo life sciences, NY, USA), 5μM SANT-1 (Cat No. 1974, Tocris Biotechnie, Bristol, UK), 10mM nicotinamide (NA), (Cat No. N0636, Sigma-Aldrich, Saint Louis, MO, USA), and 10μM retinoic acid (RA), (Cat No. R2625, Sigma-Aldrich, Saint Louis, MO, USA); and from day 8–12 also 1μM Trichostatin (TSA) ready-made solution (5mM), (Cat No. T1952, Sigma-Aldrich, Saint Louis, MO, USA). Total glucose concentration in the medium is 10mM. Stage-2 (Day 12–30): RPMI-1640 medium (Cat No. 11875093, Gibco, Grand Island, New York) with 0.05% BSA (Cat No. A9418, Sigma-Aldrich, Saint Louis, MO, USA), 1 μM T3 agonist (Cat No. 64245,Calbiochem, Millipore, CA, USA), 5μM ALK5-II Inhibitor (Cat No.ALX-270-445, Enzo life sciences, NY, USA), 10mM NA (Cat No.N0636, Sigma-aldrich, Saint Louis, MO, USA) and 50ng/ml of IGF-II (Cat No.292-G2, R&D systems, MN, USA). Differentiation medium was changed every other day until day 30 and organoids were maintained in a 37°C, 21% O_2_, 5% CO_2_ incubator. On day 12 and 30 organoids were collected for qRT-PCR. For immunostaining, organoids were fixed with 4% PFA overnight at 4°C and subsequently, stored in 70% ethanol at 4°C until embedding.

### Pancreatic organoid differentiation from hESCs

For hESC differentiation to pancreatic β-cells, we followed the protocol described earlier by our group with minor adaptations [18]. On day 30 cells were harvested for qRT-PCR.

### RNA extraction, cDNA synthesis and gene expression

Total RNA was purified using the GenElute^™^ Mammalian Total RNA Miniprep Kit (Cat No. RTN350, Sigma-Aldrich, Saint Louis, MO, USA) and ZR RNA MicroPrep (Cat No. R1061, Zymo Research, CA, USA). CDNA was generated using 0.5–1μg of RNA with SuperScript® III First-Strand Synthesis SuperMix for qRT-PCR kit (Cat No. 11752050, Invitrogen, CA, USA) and qRT-PCR was performed on a ViiA™ 7 Real-Time PCR System with 384-well plate (Cat No. 4453536, Applied Biosystems, Carlsbad, CA, USA) with a Platinum® SYBR® Green qPCR SuperMix-UDG w/ROX (Cat No. 11744500, Invitrogen, CA, USA) and primers mix at final concentration of 250nM. Gene expression (Cycle threshold) values were normalized based on the *PPIG* (peptidylprolyl isomerase G) housekeeping gene and the Delta CT calculated. Gene specific primers, purchased from IDT technologies, Leuven, Belgium were designed to distinguish between transgene (CDS-IRES) and endogenous (CDS-3’UTR or Exon-Exon spanning) gene expression. Gene expression graphs or heat maps were represented relative to the housekeeping gene *PPIG* in log scale. Heat maps were generated using Gene E software (Broad Institute of MIT and Harvard, Cambridge, MA, USA). The efficiency of primers was tested by serial dilution of cDNA and by calculating coefficient of regression (R2). An efficiency of 95‐105% with an R2≥ 97% was considered as good (see S2–S5 Tables for list of all qRT-PCR primers used in this study).

### Cell viability staining

The LIVE/DEAD® Viability/Cytotoxicity Kit (Cat No. L3224, Molecular probes, Eugene, USA) was used according to the manufacturer recommendations. Stained organoids were observed under inverted fluorescent microscope (Axiovert, Zeiss). Live and dead cells appear as green and red, respectively.

### Albumin secretion assay by ELISA

Albumin in culture supernatants and blood of grafted mice were measured using the Human Albumin ELISA Quantitation Set (Cat No.E80-129, Bethyl Laboratories, TX, USA) and the ELISA Starter Accessory Kit (Cat No. E101, Bethyl Laboratories, TX, USA) as per manufacturer’s recommendations. Absorbance was measured at 450nm using Victor 3 plate reader (Model No. 1420–041, PerkinElmer, MA, USA). The results obtained were normalized with cell number.

### Human C-peptide ELISA assay

Secretion of human C-peptide in serum from mice grafted with iENDO cells was analysed using the ultrasensitive human C-peptide ELISA kit (Cat No. 10-1141-01, Mercodia AB, Uppsala, Sweden) as per manufacturer’s recommendations.

### Immunostaining of organoids and *in vivo* grafts

14TF iENDO-hepatocyte organoids and iENDO-endocrine pancreatic organoids were fixed with 4% paraformaldehyde (PFA) (Cat No. P6148, Sigma-Aldrich, Saint Louis, MO, USA) overnight at 4°C and subsequently washed with 1X PBS and stored in 70% ethanol at 4°C. For embedding, fixed organoids were encapsulated in 2% agarose gels and stored in 70% ethanol at 4°C until embedding. Fixed iENDO kidney grafts and 3D hepatic and pancreatic organoids were processed using the Excelsior tissue processor and paraffin embedded using the HistoStar (Thermo Scientific). Some paraffin embedded sections were stained with Hematoxyline & Eosin staining. For other sections, immunostaining was performed after melting the paraffin and rehydrating the sections, which were then washed with PBS + 0.2% Triton-X-100 (PBST) for 5 minutes. Following antigen retrieval (Dako Target Retrieval Solution (1x)) sections were permeabilised with 0.2% PBST for 5 minutes. Undifferentiated 14TF iENDO cells cultured on coverslips in 12 well plate were fixed with 4% PFA and permeabilised with 0.2% PBST), blocked with 0.2% PBST + 5% normal donkey serum, and stained overnight at 4°C with primary antibodies and respective isotype controls in Dako diluent. Slides were washed with 1xPBST three times. Immune complexes were detected by incubation with a AlexaFluor AF488 (Green) and AF555 (Red) (1:500) coupled to secondary antibodies for 30 min at room temperature. The nuclei were visualized using Hoechst or DAPI (1:2,000). After 3 washes, slides were mounted with prolong Gold mounting medium. The signals were detected with a Nikon Eclipse Ti-S and Axioimager.Z1 microscope (Carl Zeiss). Identical exposure times were used for isotype and specific antibodies (See S6–S8 Tables, for list of primary, secondary, isotype antibodies). (Note: For cell surface protein CXCR4, stained with anti-human CXCR4 conjugated with PE antibody (1:100), cell permeabilisation and secondary antibody step was not followed).

### hMAPC cell surface maker analysis by flow cytometry

hMAPC cells were detached with 0.05% trypsin-EDTA and washed with 1xPBS containing 3%FBS. After washing, between 1-2x10^5^ cells were incubated for 20min in the dark at room temperature (RT) with conjugated antibodies or isotype controls (1μg/ml). A list of primary monoclonal antibodies can be found in S9 Table). 7-AAD was used as a marker to exclude dead cells from the analysis. All cells were analyzed on a FACScanto flow cytometer (BD Biosciences) with at least 10,000 recorded events. Data were analyzed with FlowJo Sofware (Tree Star, Ashland, OR, USA).

### Statistical analysis

Comparisons between two groups were analysed using an unpaired 2-tailed Student’s t-test. P-values < 0.05(*), < 0.01(**), <0.001(***) were considered significant. Data are shown as mean and error bars represent standard error of mean (SEM) of minimum three independent experiments. All results were analysed using GraphPad prism 6 software.

## Results

### 14TFs can reprogram human MAPCs into induced endodermal progenitor (iENDO) cells

**Fig 1:**
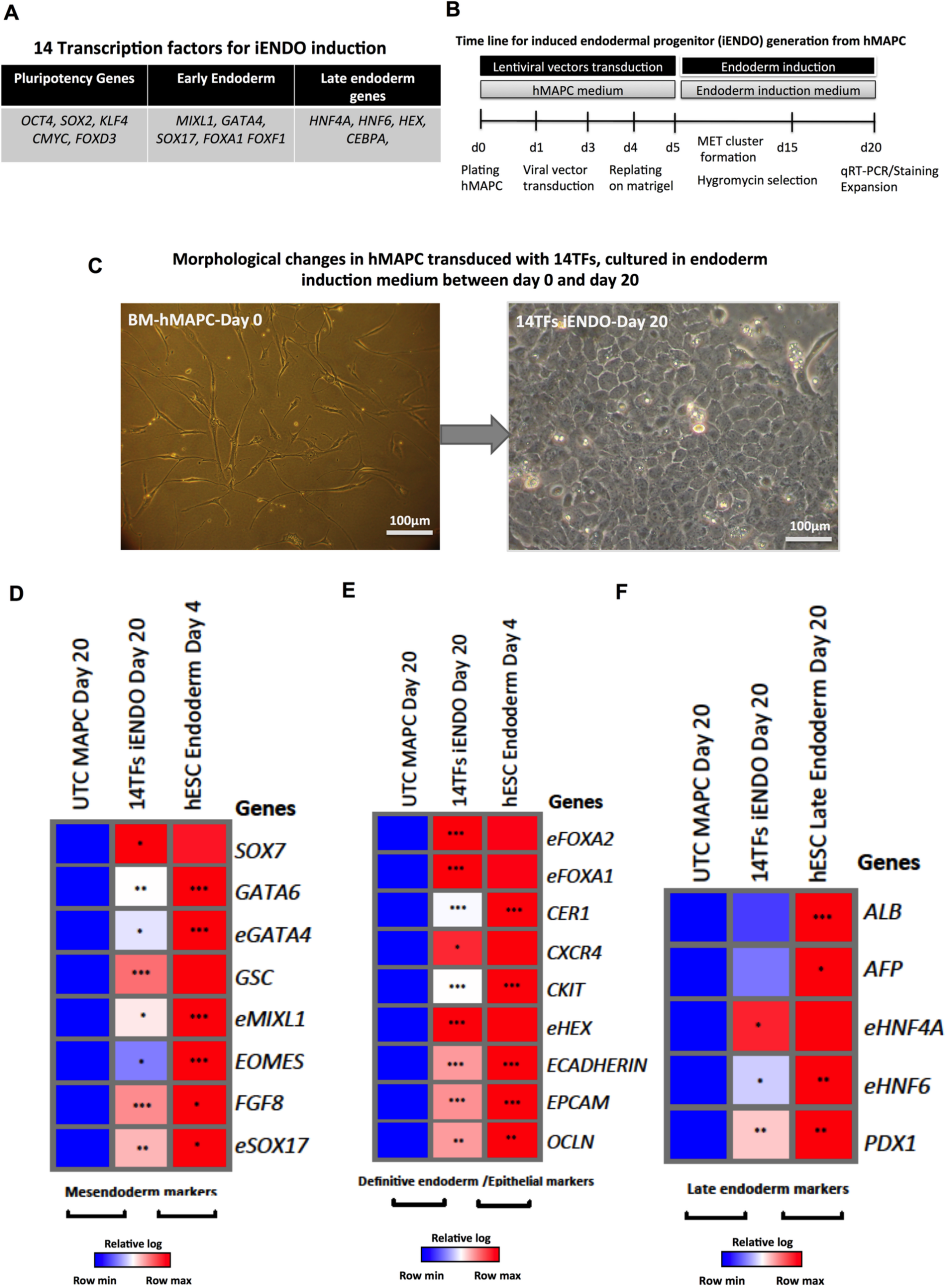
Generation of 14TF iENDO cells from hMAPC. A) Selected 14TFs for induction of iENDO cells from hMAPC B) Protocol for iENDO generation from hMAPC. C) Morphological changes of 14TF transduced hMAPC from day 0 to day 20 after transduction (scale bar: 100μm). (D-F) Relative gene expression (to *PPIG*, log scale) in day 20 14TF iENDO cells represented as a heat-map for mesendoderm (D), definitive endoderm and epithelial marker genes (E) and late endoderm marker genes (F) compared with untransduced hMAPCs, and hESC derived endodermal progenitors (d4) or mature endodermal cells (d20). All data represent mean of three independent experiments. *p<0.05, **p<0.01 and ***p<0.001 determined by unpaired 2-tailed Student’s t-test.

Initially, we transduced human MAPCs with 16 selected TFs, including the pluripotency TFs, *OCT4*, *SOX2*, *KLF4*, *CMYC* (OKSM) and TFs known to be important in mesendoderm specification (S1A Fig). Cells were maintained in endoderm differentiation medium with Activin-A/Wnt3a/BMP4 on Matrigel coated dishes from day 4 onwards (Fig 1B). On day 4, all TFs were highly expressed except for *CEBPα* (S1B Fig). Between day 9 and 20, we observed morphological changes from a mesenchymal morphology to clusters of cuboidal cells in the transduced cells, but not in untransduced or PLVX-eGFP transduced cells (S1D Fig).

On day 20 cells were harvested and transcripts for endogenous mesendoderm and as well as definitive and late endodermal marker genes assessed by qRT-PCR and compared with untransduced hMAPC cultured under the same conditions and hESCs differentiated to definitive endoderm cells (day 4) and hepatocyte-/endocrine pancreatic-like cells (day 20). For genes that were part of the pool of transduced TFs, primers were used that only detect endogenous transcripts and not the transduced transcripts (denoted by an “e” in front of the name of the gene (e.g. *eGATA6*). We found a significant induction of several mesendoderm (S1E Fig), definitive endoderm and epithelial cell marker genes (S1F Fig). Of note, the hepatic endoderm marker genes *ALB* and *AFP* (S1G Fig) were also induced, which could be consistent with the known role of *FOXA2* and *HNF1α*, as key regulators of hepatocyte differentiation [23, 41, 42].

Therefore, we repeated the studies without using the *FOXA2* and *HNF1α* viral vectors. Human MAPCs were transduced with 14 TFs (Fig 1A) and maintained as described above (Fig 1B). Between day 9 and 20, we again observed morphological changes from a mesenchymal morphology to clusters of cuboidal cells (Fig 1C). On day 4, all TFs were expressed (S1C Fig). When MAPCs were transduced with only OSKM in endoderm induction medium, clusters of cuboidal cells were observed, which were not expandable and did not express endodermal transcripts (S2A Fig). When OSKM was omitted from the 14TFs no cuboidal cells were observed (S2B Fig).

**Fig 2:**
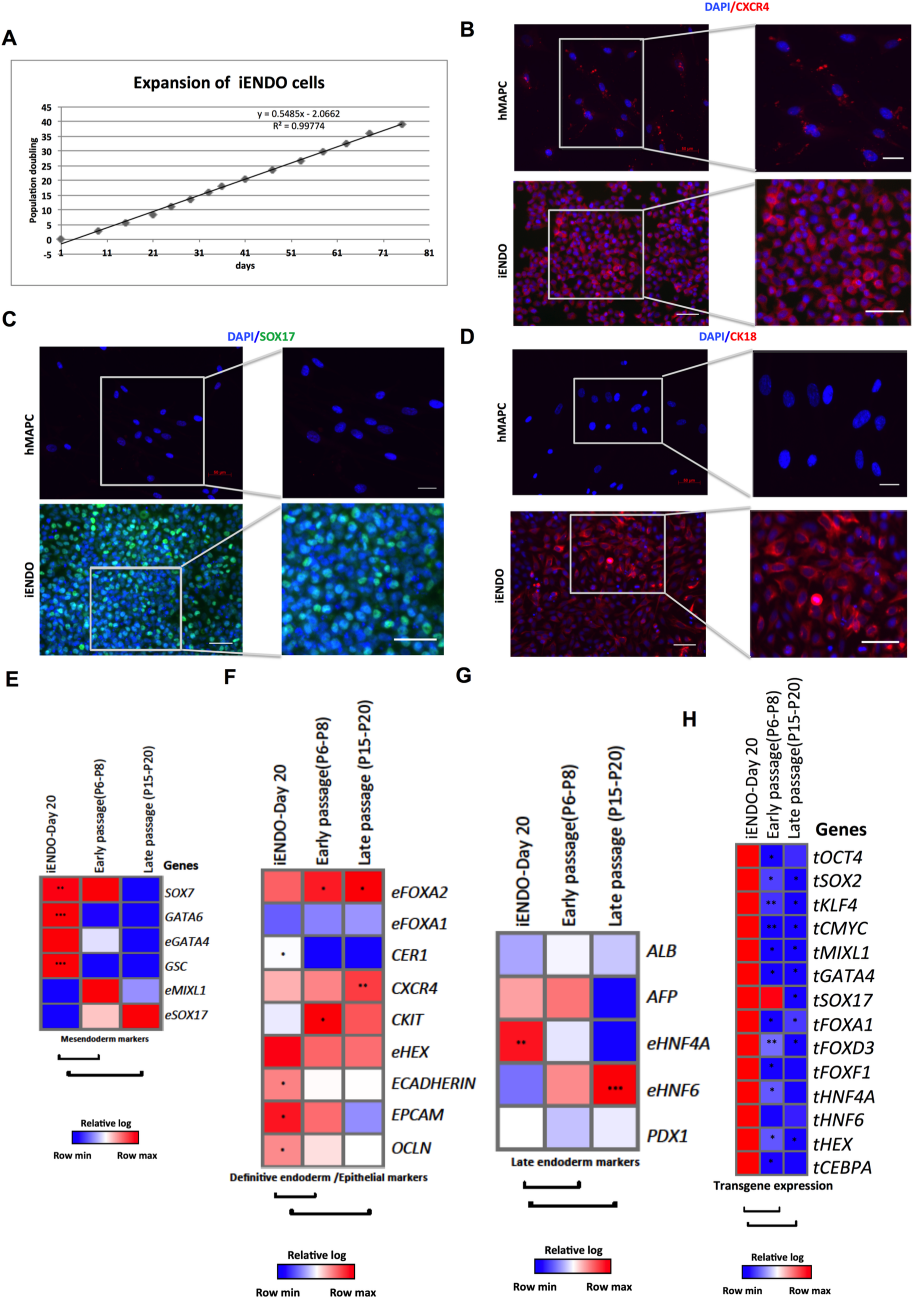
14TF iENDO cells are expandable long-term *in vitro*. A) Expansion curve of 14TF iENDO cells shown as days in culture (horizontal axis) vs. the number of population doublings (PDs) (vertical axis). B) Immunostaining analysis of CXCR4 (C) SOX17 and CK18 (D) and protein expression of 14TF iENDO cells, at passage 4 (magnification 20x, scale bar: 50μm and 40x, scale bar: 100μm), representative sample of n = 3. (E-G) Relative gene expression (to *PPIG*, log scale) in 14TF iENDO cells at 6–8 and 15–20 passages represented as a heat-map for mesendoderm (E), definitive endoderm and epithelial marker genes (F) and late endoderm marker genes (G) compared with day 20 iENDO cells. H) Relative gene expression (to PPIG, log scale) represented as a heat-map of transgene expression in 14TF iENDO cells on day 20, P6-P8, and P15-P20. All data represent mean of three independent experiments. *p<0.05, **p<0.01 and ***p<0.001 determined by unpaired 2-tailed Student’s t-test.

On day 20, reprogrammed cells were harvested and transcripts for the markers described above assessed by qRT-PCR. We again found significant induction of many mesendoderm, definitive endoderm and epithelial marker genes (Fig 1D and 1E) compared to untransduced MAPC cultured in the same medium. Levels of mesendoderm genes were signifcantly lower than in day 4 ESC endodermal progeny and levels of definitive endoderm genes approached levels of hESC-endoderm committed cells, and as hoped for, expression of more mature hepatic and pancreatic endoderm genes remained well below levels found in ESC progeny allowed to further mature to hepatocytes and pancreatic endoderm. The endogenous pluripotency genes *eOCT4* and *NANOG* were not activated (data not shown). iENDO cells also stained positive for endodermal markers (CXCR4: 84% ± 9%, SOX17: 90% ± 16%; CK18: 85% ± 15%; (Fig 2B–2D) and respective isotype controls (S2C–S2E Fig). Most importantly, the late hepatic endodermal markers, *ALB* and *AFP* were expressed but significantly less higher level when compared with 16TF-iENDO cells (Fig 1F), which was confirmed by immunostaining (S2F–S2H Fig), Also, iENDO cells express trangene OCT4 at protein level (S2I Fig). We assessed the purity of hMAPCs by cell surface marker expression using flow cytometry (S3A and S3B Fig). We found hMAPC to be negative for the hematopoietic markers, CD45, CD34, CXCR4, and CKIT; the endothelial marker CD31; as well as the CD140a, CD140b, ALP, HLA-class-II, CD271 markers (S3A Fig); and positive for CD44, CD90, CD105, HLA-class 1, CD73, CD146 (S3B Fig). This expression profile is similar to our previous publication [34]. In contrast, in 14TFs iENDO only minimal expression levels of the hMAPC cell surface markers CD 73 and CD105 were detected (S4A and S4B Fig).

### 14TF iENDO cells can be expanded for at least 20 passages without significant loss of their endodermal progenitor phenotype

The rationale for making iENDO cells, rather than direct reprogramming of somatic cells to mature endodermal cells, was to create an expandable population of cells. Therefore 14TF iENDO cells were passaged in endoderm induction medium with 50ng/ml Activin-A and 25 ng/ml Wnt3a for 6 to 8 passages on Matrigel-coated dishes. In addition we expanded the iENDO cells from passage 6/8 to 20 in a less complex and less expensive medium, namely hMAPC medium on 0.1% gelatin-coated dishes (Fig 2A). We reevaluated expression of the mesendoderm, definitive endoderm, epithelial and late endoderm markers after 6–8 and 15–20 passages. Both after 6–8 and 20 passages, most endogenous transcripts remained unchanged compared to day 20 iENDO cells, even if the definitive endoderm transcripts, *SOX17*, *eFOXA2*, *CXCR4*, *CKIT*, *CXCR4*, and *eFOXA1*, as well as the hepatoblast transcript *HNF6* were increased, while *eHNF4a* decreased (Fig 2E–2G). Importantly, more mature pancreatic and hepatocyte transcripts remained very low. In addition, transcript levels of all transgenes were significantly decreased in 6–8 and 20 passage iENDO cells (Fig 2H). These studies demonstrated that it is possible to create a population of endodermal progenitor cells from hMAPCs that can be expanded at least 20 passages, using a relatively non-expensive medium with a stable transcriptional profile. Genomic stability of early (P4) and late passage (P20) 14TFs iENDO shown that there is no significant genome wide aberrations observed (S5A and S5B Fig).

### Grafting of 14TF iENDO cells *in vivo*

**Fig 3:**
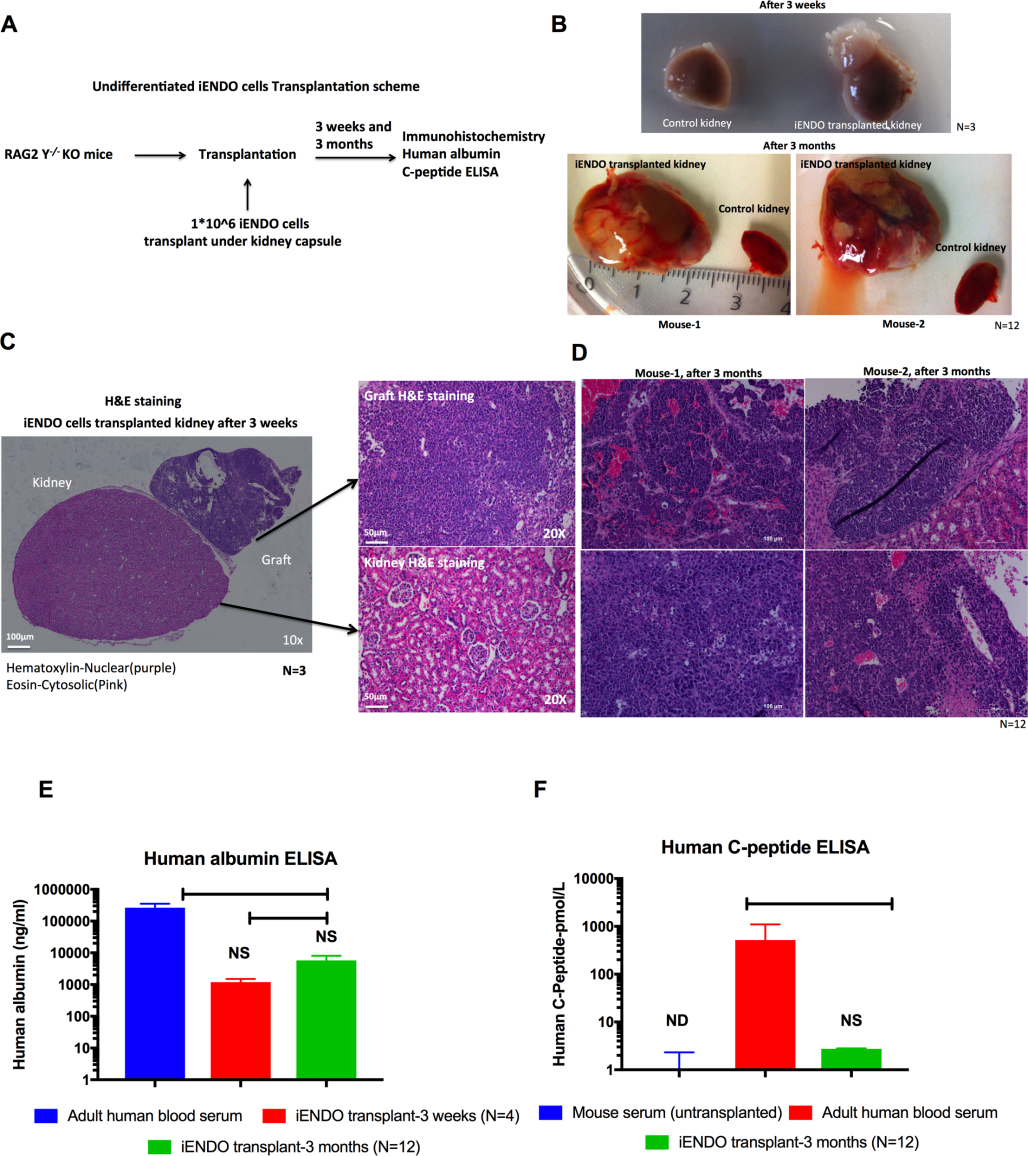
*In vivo* transplantation of 14TF iENDO cells under the kidney capsule forms endodermal tumors but that differentiate into hepatoblasts and pancreatic endocrine like cells. A) Schematic representation of transplantation of undifferentiated iENDO cells under the kidney capsule of RAG2^-/-^ γc^-/-^ mice. B) Morphology of control kidney (half portion shown) and iENDO cell grafted kidney after three weeks (N = 3) and after three months (N = 12). C) H&E stained sections of iENDO graft (10x and 20x magnification) 3 weeks after transplantation (scale bar 50μm) (N = 3). D) H&E stained sections of iENDO graft (20x magnification) 3 months after transplantation (mouse 1 and mouse 2)(scale bar 100μm) Representative for (N = 12) mice. E) Human albumin quantification by ELISA from iENDO cell grafted mice 3 weeks (N = 3) and 3 months (N = 12) after transplantation, untransplanted mouse serum and human blood serum. F) Human C-peptide by ELISA (untransplanted mouse serum, adult human blood serum and 14TF iENDO grafted mouse 3 months after transplantation (N = 12). *p<0.05, **p<0.01 and ***p<0.001 determined by unpaired 2-tailed Student’s t-test. ND- Not determined, NS- Not significant.

To test their *in vivo* differentiation capacity, we transplanted one million 14TF ENDO cells (passage 6) under the kidney capsule of BALBC Rag2^-/-^ γc^-/-^ mice in 30μl of PBS (Fig 3A). After grafting, the weight and glucose levels of the mice were regularly assessed. Three mice were sacrificed after 3 weeks (N = 3) and the other (N = 12) were maintained untill 3 months after grafting. Unfortunately, of the last group, one mouse died in the 12^th^ week. In all mice, we found that a tumor was formed surrounding the kidney. The tumor was larger at 3 months after transplantation (Fig 3B).

Graft sections were stained with Hematoxylin and Eosin to assess the graft morphology. None of the grafts had invaded into the kidney. In general, at 3 weeks (N = 3) and at 3 months (N = 12), 14TF iENDO cells-derived tumor cells were homogenous containing endodermal cells, akin to a hepatocellular carcinoma (Fig 3C and 3D). However, the graft of the 12 mice sacrificed at 3 months, showed a more heterogeneous morphology, even if most cells still appeared endodermal (Fig 3D).

We immunostained the grafts for the hepatocyte markers AFP, ALB, AAT, HNF4α and the transgene OCT4. Most cells stained positive for OCT4 after 3 weeks (S6A–S6E Fig) (N = 3) and after 3 months (N = 7), cells in the grafts stained positive for the liver markers AFP, ALB and HNF4α, but fewer for AAT (S7A–S7D Fig). In addition, many cells stained postive for OCT4 (S7E Fig). Cells in the graft also stained positive for the intestinal brush-border microvilli marker, VILLIN-C, and the Paneth cell marker, LYSOZYME (S8A Fig), while fewer cells stained positive for the pancreatic endoderm marker PDX1 (S8B Fig). One of the 3-month grafts was morphologically more heterogeneous, and contained fewer AFP, ALB and HNF4α positive cells (S9A–S9D Fig). However, clusters of cells stained positive for the pancreatic endoderm markers PDX1 (S9E Fig and S10A Fig) and NGN3 (S10A Fig), and very few also for SST (S10B Fig). Insulin- or glucagon- positive cells were not detected. In addition, cells in the graft stained positive for the neuroendocrine marker CHGA (S10C Fig), the paneth cell marker, LYSOZYME (S10D Fig) and the intestinal brush-border microvilli marker, VILLIN-C (S10E Fig). Also, many of the cells still stained positive for OCT4 (S10F Fig). In addition, mesodermal lineage markers, as an example the cardiac specification marker NKX2.5 and ectodermal lineage markers, as an example the neuronal marker β-TUBULIN-III were not observed by immunostaining (S11A and S11B Fig) or by qRT-PCR (S11C and S11D Fig). Control, non-grafted kidney sections did not stain positive for any of these markers.

We also performed qRT-PCR on grafts harvested at 3 weeks (N = 3) and at 3 months (N = 9). At 3 weeks transcript levels for *ALB*, *AFP*, *AAT* and *TTR* were significantly higher in the graft compared with the initial iENDO cell population, even if mature hepatocyte markers G*6PC*, *NTCP*, *MRP2* and *CYP3A4* were not significantly increased (S12A and S12B Fig). Likewise, after 3 months, transcript levels for *ALB*, *AFP* and *TTR*, but not *AAT*, *G6PC*, *NTCP*, *MRP2* and *CYP3A4* were significantly increased in the graft compared with the initial cell population (S12A and S12B Fig). Consistent with the immunostaining results, we also detected increased levels of the pancreatic endoderm transcripts *PDX1*, *NGN3*, *NKX6*.*1*, *NEUROD1*, *PAX4*, *SST*, but not *INS or GCG*; as well as the gut markers, *HNF1β* and *KRT19*, *CDX2* in mouse 2 (S12C–S12E Fig). Transcripts for the transgenes were similar before and after grafting, both at 3 weeks and at 3 months (S12F Fig).

We analyzed the mouse serum for presence of human albumin. After 3 weeks, human albumin levels were 1,189 ± 615.44 ng/ml (N = 4), and after 3 months 5,777 ± 7,928 ng/ml (N = 12). In comparison, human blood serum albumine levels are 265,499 ± 12,5354 ng/ml (Fig 3E), while human albumin levels in untransplanted mice were 377± 3.28ng/ml. The levels of human albumin depicted are the levels measured minus the levels found in untranplanted mouse serum. Negligible levels of human C-peptide (2.735 ± 0.06 pmol/L) were detected in the blood of (mouse 1&2), 3 months after grafting (Fig 3F), while no c-peptide was detected in the blood of mice 3 months after grafting. c-Peptide levels in human serum were 518.63 ± 584.62 pmol/L.

These studies demonstrate thus that 14TF iENDO cells can differentiate *in vivo* to multiple endodermal lineages, including hepatoblast, pancreatic endoderm, as well as gut endoderm like cells.

### 14TF iENDO cells can differentiate into hepatocyte-like cells

**Fig 4:**
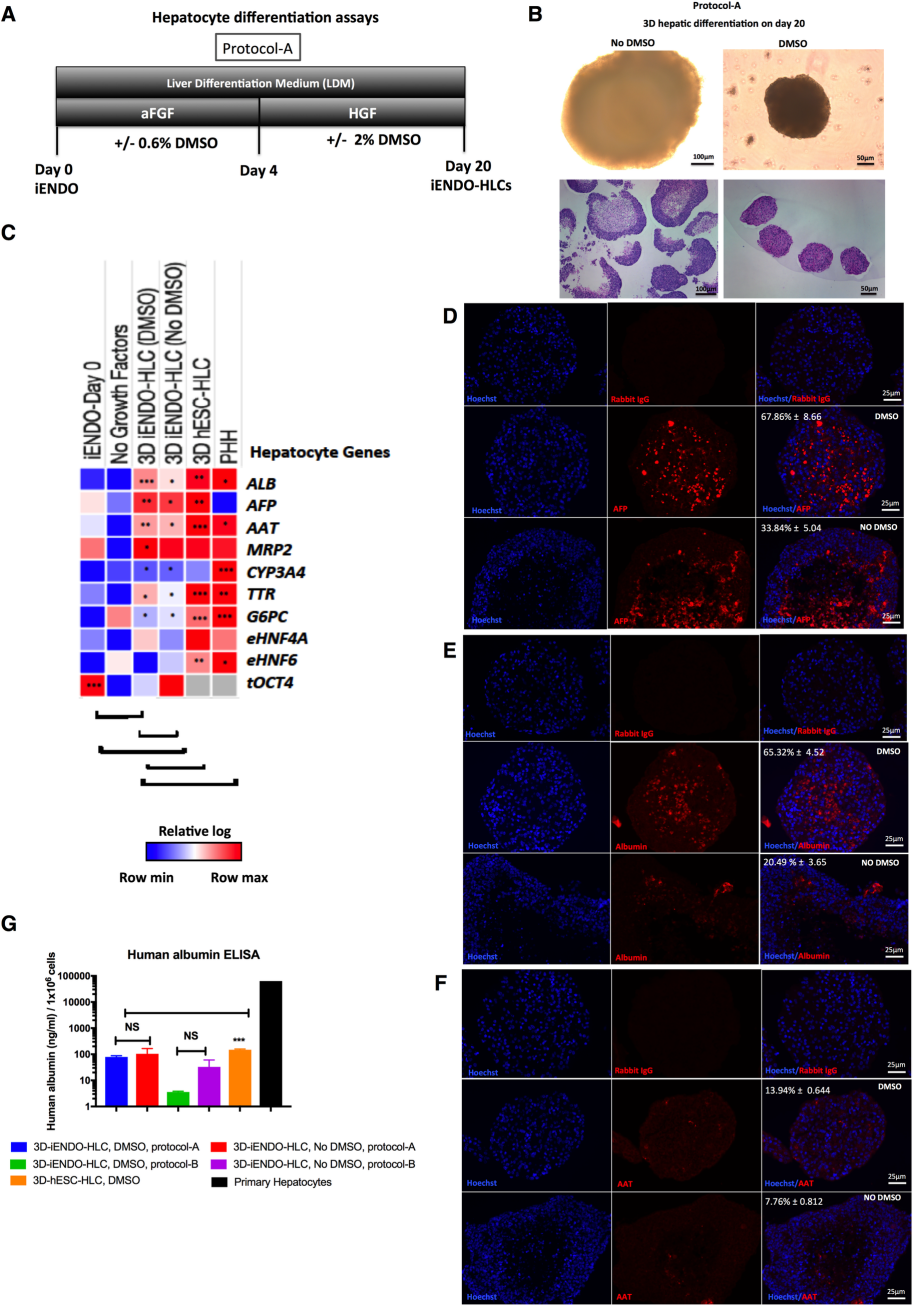
14TF iENDO cells differentiate into hepatocyte like cells. A) Protocol time-line for hepatocyte differentiation in 3D from iENDO cells protocol A with or without DMSO. B) Morphology of iENDO differentiated organoids at day 20 with and without DMSO by bright field and H&E staining (N = 3). C) Relative gene expression (to *PPIG*, log scale) in day 20 iENDO-HLCs ± DMSO represented as a heat-map for hepatocyte markers compared with hESC-HLCs organoids (d30) and PHHs (N = 3). D-F) Immunostaining for AFP, ALB and AAT on day 20 iENDO organoids ± DMSO with respective isotype controls. Scale bar 25 μm (representative example of N = 3). G) Albumin secretion in supernatants of day 20 iENDO organoids ± DMSO, hESC day 30 organoids and primary hepatocytes (PHH) (N = 3). Error bars represents standard deviation of three independent experiments. *p<0.05, **p<0.01 and ***p<0.001 determined by unpaired 2-tailed Student’s t-test. NS- Not significant.

As spontaneous differentiation occurred from iENDO cells to cells with hepatoblast features *in vivo*, we assessed if iENDO cells could be directly differentiated *in vitro* to cells with mature hepatocyte features, using methods developed in the lab for hPSCs[40]. As iENDO cells have an endodermal phenotype, we started differentiation from day 8 (hepatic endoderm specification) or day 12 (hepatoblast maturation) of the hPSC protocol onwards (protocol A vs. B, respectively (Fig 4A and S13A Fig). To determine if there are morphological differences between iENDO derived hepatocyte organoids cultured following protocol-A (day 20) or following protocol-B (day 16) and cultured with or without DMSO, pictures were taken at the end of each protocol. Not many differences were observed between organoids of the two protocols. However, organoids cultured with DMSO appeared smaller than the spheroids cultured without DMSO (Fig 4B and S13B Fig). No DMSO condition cultured organoid core seems to have dead cells compare to the DMSO by H&E Staining (Fig 4B and S13B Fig). An additional variable was addition of DMSO, as others[11, 15, 43, 44] and we have demonstrated that this improved the overall differentiation of PSCs to hepatocyte-like cells (HLCs)[40]. Initial studies demonstrated that the highest transcript levels for *ALB*, *AFP*, *AAT*, *MRP2*, *CYP3A4*, *TTR*, *G6PC*, *eHNF4α* (Fig 4C) were detected when cells were differentiated using protocol A with addition of DMSO compare to the protocol-B (S13C Fig). Therefore, we chose protocol-A + DMSO for further evaluation and comparison with ESCs differentiated as 3D organoids.

Expression levels *of ALB*, *AFP*, *AAT*, *MRP2* and *TTR* transcripts were ± 1 log lower than d20 differentiated iENDO compared with day 30 3D ESC-HLCs (differentiated as described[45], until day 20 and as organoids until day 30)(Fig 4C). Transcripts for *eHNF4α*, and *eHNF6*, *CYP3A4* and *G6PC* in 3D iENDO differentiated organoids were comparable with 3D-ESC-HLCs (Fig 4C). Addition of DMSO to either iENDO or hESC differentiation improved differentiation (Fig 4C). Pancreatic endocrine gene transcript levels were not induced under hepatic differentiation conditions (S14A and S14B Fig). When compared to primary human hepatocytes (PHHs), 3D iENDO and 3D hESC hepatocyte-like cells continued to express *AFP*, while transcripts for mature hepatocytes were significantly lower compared with PHHs (Fig 4C). Transgenic *tOCT4* transcripts remained detectable in iENDO-HLCs, albeit at decreased levels compared to undifferentiated iENDO cells (Fig 4C). As negative control, iENDO cells were cultured as organoids without growth factors and small molecules (Fig 4C and S13C Fig), which did not induce increased expression of hepatocyte transcripts.

Immunostaining studies revealed that 3D-iENDO-HLC organoids, differentiated with DMSO, contained 67.86% ± 8.66 AFP^+^, 65.32% ± 4.52 ALB^+^, and 13.94% ± 0.644 AAT^+^ cells. The percentage AFP^+^ and ALB^+^ cells was significantly higher than in 3D organoids differentiated without DMSO and levels were comparable with hESC-HLC 3D organoids (Fig 4D–4F).

To study the function of iENDO-derived HLCs, we measured albumin secretion by ELISA: supernatants of 3D iENDO-HLCs contained 78.737 ± 8.70 ng/ml ALB (n = 3) in the presence of DMSO and 104.5 ± 61.68 ng/ml without DMSO (n = 3), while supernatants of 3D ESC HLCs cultured with DMSO contained 149.17 ± 9.93 ng/ml ALB (n = 4). Thus levels were similar for 3D iENDO and 3D hESC-HLCs, but significantly lower than in PHHs(Fig 4G).

Thus, consistent with the *in vivo* data, iENDO cells can be guided towards HLCs *in vitro* even though the differentiated cells are less mature than PHHs.

### 14TF iENDO cells can differentiate into pancreatic endocrine cells

**Fig 5:**
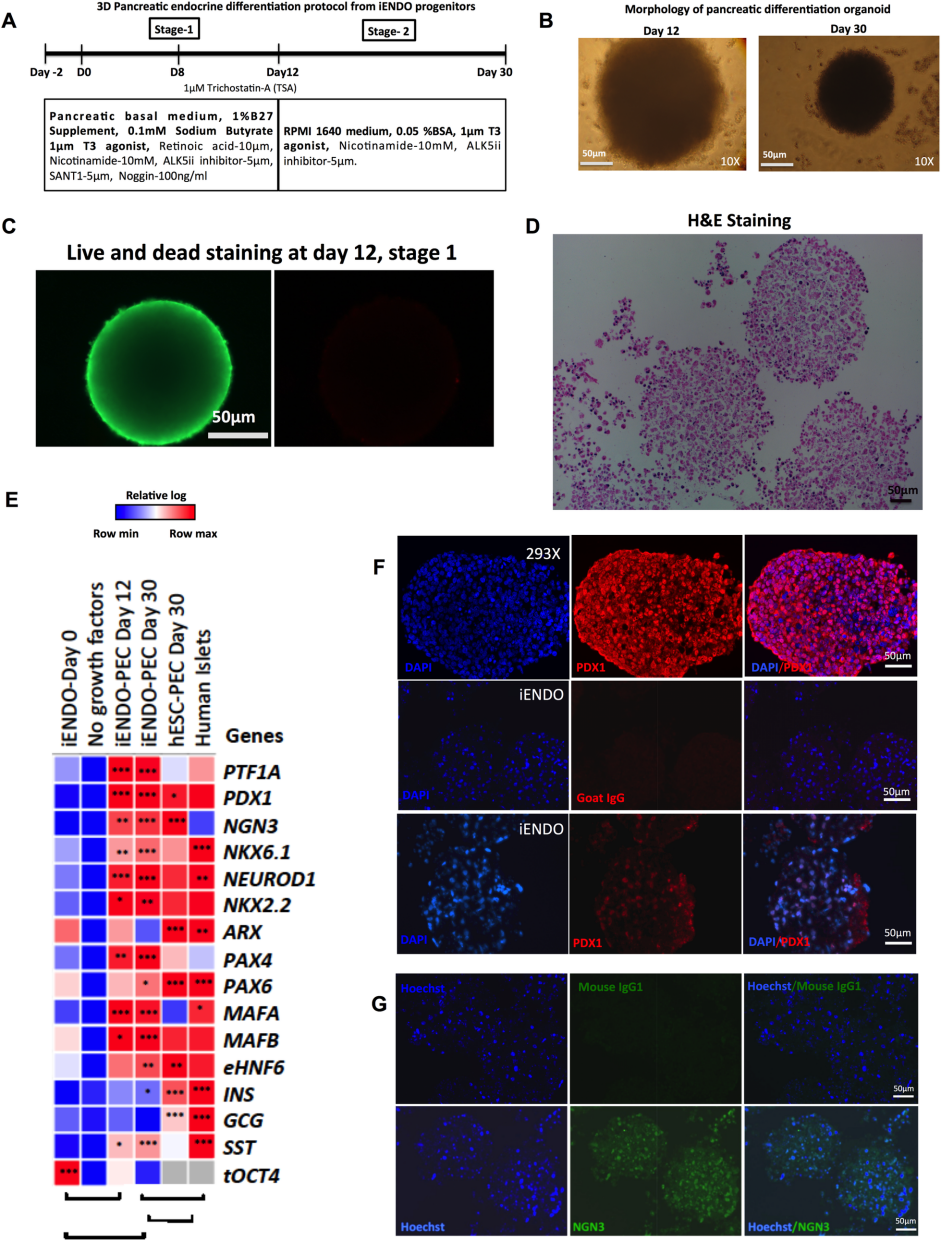
Generation of pancreatic endoderm and endocrine progenitor-like cells from iENDO. A) Protocol time-line for pancreatic endocrine differentiation from iENDO cells. B) Morphology of 3D aggregate at day 12 and day 30 of differentiation, 10x magnification (scale bar 50μm). C) Live/Dead staining of 3D aggregates at day 12 of differentiation (Live-green, Red-dead) (scale bar 50μm). D) H&E staining of day 12 iENDO pancreatic organoids. E)Relative gene expression (to *PPIG*, log scale) analysis represented in a heat-map of pancreatic endocrine marker 14TFs iENDO day 0, day 12 iENDO pancreatic organoids, day 30 3D iENDO pancreatic organoids and day 30 hESC pancreatic organoids compared with primary human Islets. All data represent mean of three independent experiments. F) Immunostaining for PDX1 (20X) in 293x transduced PDX1 (top) and day 12 iENDO pancreatic organoids (bottom) (scale bar 100μm, representative of N = 3) with respective isotype controls. F) Immunostaining for NGN3 (20X) in day 12 iENDO pancreatic organoids with respective isotype controls (scale bar 50μm, representative of N = 3). *p<0.05, **p<0.01 and ***p<0.001 determined by unpaired 2-tailed Student’s t-test.

We also assessed if 14TF iENDO cells could be differentiated towards endocrine pancreatic cells. 14TF-iENDO cells (passage 6–12) were cultured as organoids with combinations of growth factors and small molecule epigenetic modifiers known to induce endocrine pancreas differentiation as described in (Fig 5A)[18, 21, 22]. We observed compact spheroids as early as 2 days after plating the cells. The morphology of spheroids on day 12 and day 30 of the protocol is shown (Fig 5B). There was not much difference observed between spheroids on day 12 and day 30, except the size of the organoid which was smaller at day 30 than day 12. On day 12, Live/Dead and H&E staining demonstrated that cells in the organoids were viable (Fig 5C and 5D). In parallel, hESCs differentiated into pancreatic endocrine cells by using the protocol described in our group previously[18].

On day 12 (stage 1) and day 30 (stage 2) the expression of pancreatic marker genes was assessed by qRT-PCR (Fig 5E), and compared with undifferentiated 14TF-iENDO cells (day 0), hPSCs differentiated to pancreatic endocrine cells at day 30, and primary human islets. We detected a significant increase in gene expression of *PTF1A*, *PDX1*, *NGN3*, *NKX6*.*1*, *NEUROD1*, *NKX2*.*2*, *PAX4*, *MAFA*, *MAFB*, *EHNF6* and *SST* but not for INS and *GCG*, nor *NKX6*.*1* (master regulator β-cells) or *ARX* (master regulator α-cells) (N = 5) (Fig 5E). When compared with hPSC progeny, transcript levels in day 30 iENDO progeny were similar to those of hPSC progeny, except that *INS and GCG*, *ARX*, *PAX6* expression was significantly higher in hPSC progeny. When compared with primary islets, *INS*, *GCG and SST* expression in both iENDO and hESC progeny was significantly lower, whereas endocrine pancreatic genes, including *NGN3*, were significantly higher expressed in iENDO and ESC progeny (Fig 5E). Also, hepatocyte marker transcripts were not induced in iENDO derived pancreatic progeny (S14C and S14D Fig). Following differentiation of iENDO cells, the *OCT4* transgene remained expressed. When iENDO cells were cultured without growth factors and small molecules as organoids, pancreatic transcripts were not induced (Fig 5E).

Day 12 organoids were encapsulated in 2% agarose gel and paraffin embedded sections immunostained for the pancreatic endoderm markers. PDX1 and NGN3 were expressed (Fig 5F and 5G lower image).

Thus, consistent with the in vivo data, iENDO cells can be guided towards endocrine pancreatic cells *in vitro*, even though not to mature β- or α-cells.

## Discussion

In this study, we demonstrated that hMAPCs can be reprogrammed into a population of iENDO cells using a combination of 14TFs and culture medium containing Activin-A, Wnt3a and BMP4. iENDO cells can subsequently be expanded for at least 20 passages using a relatively inexpensive medium and can differentiate *in vivo* and *in vitro* into multiple endodermal cell types.

Reprogramming hMAPCs to iENDO cells was dependent on the presence of OSKM as well as the growth factors Activin-A, Wnt3a and low doses of BMP4. Indeed, removal of either of these components from the reprogramming method failed to induce the generation of epithelioid clusters and induction of endogenic mesendoderm and definitive endoderm transcripts. This is in line with studies described by Funa S et al. demonstrating that interaction between the TF *OCT4*, the Nodal effectors SMAD2/3, and Wnt effector β-catenin is required to activate primitive streak gene expression by co-operatively binding to the chromatin upstream of primitive streak gene loci[46]. Moreover, Loh et al. demonstrated that low concentrations of BMP4 and Wnt initially specify the anterior primitive streak[47], whereas Li et al. found that BMP4 combined with OSKM can activate epithelial markers such as *E-CADHERIN*, *EPCAM*, *OCLN* (OCCULUDIN)[48]. Recently, Li et al. and Zhu et al. also demonstrated that definitive endoderm- like cells expressing *SOX17* and *FOXA2* could be generated from mouse and human fibroblasts by transient overexpression of OSKM combined with a cocktail of epigenetic molecules. These endodermal cells could then subsequently be converted to pancreatic β-cells and hepatocytes, even if knockdown of p53 was required to create the mature progeny from human endodermal cells [25, 33, 49].

We also assessed whether, aside from OSKM, the other 10 TFs used in the 14TF used to reprogram hMAPCs to iENDO cells were required. To identify the required complement of TFs for reprogramming MAPCs into iENDO, we removed single TFs from 14 transcription factors and assessing creation of cells with iENDO features. When we removed *OCT4*, *SOX2*, *KLF4*, *SOX17*, or *MIXL1*, *GATA4*, *HNF6* from the 14TF pool, the efficiency of iENDO formation decreased, as the resultant cell population did not express the same levels of endogenous definitive endoderm transcripts and the cells could not be expanded (data not shown).

When iENDO cells were grafted under the kidney capsule of immunocompromised mice, we found differentiation solely to endodermal cell, including cells with hepatoblast and endocrine pancreatic characteristics and to a lesser extent cells with intestinal cell characteristics, but not ectodermal or mesodermal positive cells. The reason why, in one mouse assessed at 3 months, iENDO cells differentiated predominantly to hepatoblast-like cells, and in the other mouse, to pancreatic and gut endoderm is currently not clear. However, the iENDO cells formed progressively increasing tumors, which did not invade the kidney. These data are consistent with a study by Seguin et al. wherein *SOX17* was overexpressed in hESCs, which lead to the formation of tumors containing both mesodermal and endodermal cell types[50]. As is the case of iENDO cells, the *SOX17* overexpressing hESCs also still expressed *OCT4*, which we believe may be responsible for the continued proliferation *in vivo*. However when Cheng et al. showed that when CXCR4/CKIT enriched endodermal progenitors or human foregut stem cells derived from hPSCs were transplanted in mice, formation of mature endodermal tissues or foregut endodermal cells, respectively, was observed without formation of a tumor[51, 52]. In both studies, *OCT4* expression was no longer detected in the PSC-derived endodermal progenitor cells that were grafted.

Of note, the hepatoblast-like cells in the iENDO progeny secreted significant amounts of human albumin. When compared with published reports wherein HLCs derived from hESC or hiPSC, albumin levels in the blood of our mice grafted with iENDO cells (±1200 ng /ml after 3 weeks, and >5000 ng/ml after 3 months) appeared to be similar or even higher, except in the mouse wherein the graft had differentiated towards predominantly pancreatic and gut endoderm cells. For instance, Duan et al. reported human albumin levels in the grafted mouse blood of 4–35 ng/ml after 3 weeks[53]; Yusa et al. 40-100ng/ml at 4–5 weeks after grafting; Basma et al. 1000–2000 ng/ml human albumin after 75 days[43]; and Takebe et al., 600-1700ng/ml after 40 days[54]. Albumin levels detected in mice grafted with induced hepatocytes (iHEPs) were reported to be as high as 300 μg/ml in the Du et al. study after 7 weeks[24], but only 400 ng/ml in the Huang et al. study[23]. Differences in the number of cells that secrete albumin *in vivo* as well as the location of the graft, as well as the mouse model used may impact the results observed. Nevertheless, except for the Du et al. studies, albumin levels detected following grafting of iENDO cells are in line with or higher than published results for HLCs derived from PSCs and iHEPs.

In line with the *in vivo* data demonstrating that iENDO cells can mature further towards different endodermal lineages, we demonstrated in directed *in vitro* differentiation studies that iENDO cells can generate hepatocyte-like cells and endocrine pancreatic cells. As iENDO cells already have an endoderm/definitive endoderm phenotype, we omitted the initial commitment step (using Activin-A and Wnt3a) commonly used to differentiate PSCs to endodermal cells towards the pancreatic or hepatocyte lineage.

iENDO progeny expressed signficantly higher levesl of numeorus hepatic genes, to levels near to ±1 log lower compared with hESC progeny. However, as has been described[19, 20], the differentiation system did not induce fully mature hepatocytes, as the iENDO progeny, like hESC-progeny, did not express mature hepatocyte transcripts (such as *G6PC* and *CYP3A4*), did not have significant CYP3A4 activity and continued to express high levels of *AFP*. As has been demonstrated by others[55, 56] and us[40] for hPSC differentiations to HLCs, addition of DMSO increased HLCs differentiation of iENDO cells.

To differentiate iENDO cells towards endocrine pancreatic cells we used a protocol adapted from Pagliuca et al.[21] and Alireza Rezania et al.[22], wherein was demonstrated that mature β-cells can be generated from hPSCs. Although pancreatic endocrine transcripts were induced by the combination of growth factors and small molecules described, and organoid formation, the master regulators for α-cell (*ARX* and *PAX6*) and β-cell (*NKX6*.*1*) remained very low, and consistent expression of *INS* and *GCG* transcripts also remained very low. When compared with hESC differentiation as we described previously[18], we found that iENDO cells differentiated less robustly. hESCs progeny expressed higher levels of the master regulators for α-cells, which was in accordance with higher transcript levels for *GCG*. Moreover, *INS* transcripts were significantly higher in hESCs progeny compared with iENDO progeny. A possible explanation for the lower degree of maturation of iENDO-derived progeny compared with hESCs progeny could be the persistent expression of the transgene *OCT4* after differentiation. When compared with primary islets, iENDO cells were significantly less mature, with retained expression of *NGN3*, and have significantly lower levels of *INS* and *GCG* transcripts as well as their upstream transcriptional regulators. Of note, *NGN3* transcripts in hESCs progeny were also still significantly higher than in mature islets.

In conclusion, we demonstrated that it is possible to reprogram hMAPCs that do not have a robust endodermal differentiation potential to a long-term expandable population of endodermal progenitor cells using TFs and growth factors. These iENDO cells can differentiate both *in vitro* and *in vivo* to multiple different more mature endodermal cell types, even if fully mature hepatocytes and β-cells could not be generated and persistent expression of the pluripotency TF, *OCT4*, may be responsible, at the same time, for the continued proliferation of the iENDO cells *in vivo*, and in part for the incomplete maturation of the iENDO cell to mature endoderm cell types. Future studies wherein *OCT4* is only transiently expressed may shed light on this. Nevertheless, the current study describes an alternative cell source, which might be a starting point for the creation of mature endodermal cells that could be considered for regenerative medicine and drug studies.

## Supporting information

S1 Fig hMAPC transduced with 16TF results in cells expressing mature hepatocyte markers, not only endodermal markers. A) Selected list of 16 transcription factors. B) Transgene expression analysis by qRT-PCR after 16TF transduction on day 4 C) Transgene expression analysis by qRT-PCR after 14TF transduction on day 4. D) Morphological changes of 14TF transduced hMAPC from day 0 to day 20 after transduction. Untransduced BM-hMAPC and PLVX-eGFP transduced BM hMAPC at day 20 did not show morphological changes (scale bar 100μm). E-G) Relative gene expression (to *PPIG*, log scale) in day 20 16TF transduced cells represented as a heat-map for mesendoderm (E), definitive endoderm and epithelial marker genes (F) and late endoderm marker genes (G) compared with untransduced hMAPCs and hESC derived endodermal progenitors (day 4) or mature endodermal cells (day 20). All data represent mean of three independent experiments.*p<0.05, **p<0.01 and ***p<0.001 determined by unpaired 2-tailed Student’s t-test.
(TIF)

S2 Fig Screening of transcription factors for iENDO generation. A) cuboidal morphology was seen when hMAPC cells were transduced with OSKM alone and endoderm induction medium from day 12 onwards. B) Cuboidal morphological changes were not observed in hMAPC transduced without OSKM (12TFs). C-E) Isotype control staining for CXCR4, SOX17 and CK 18 antibody in hMAPC and iENDO cells. F-H) 14TF iENDO stained for ALB, AFP and AAT with respective isotype controls. I) 14TF iENDO stained for OCT4 with respective isotype control. Representative for 3 independent experiments. Scale bar 100μm.
(TIF)

S3 Fig Cell surface marker expression of hMAPC by flow cytometry. A) Histograms for surface markers: CD45-FITC, CD34-FITC, CXCR4-PE, CKIT-APC, CD140a-PE, CD140b-PE, CD31-PE, ALP-APC, HLA Class-II-PE, and CD271-PE with respective isotype control. B) Histogram for surface markers: CD44-APC, CD90-FITC, CD105-APC, HLA Class1-APC, CD73-PE, and CD146-PE with respective isotype control. Representative for 3 independent experiments.
(TIF)

S4 Fig 14TFs iENDO express minimal levels if hMAPC cell surface markers after reprogramming. **A-B)** Histograms for hMAPC surface markers staining on 14TFs iENDO cells: CD73-PE and CD105-APC with respective isotype control. Representative for 3 independent experiments.
(TIFF)

S5 Fig Genomic stability of 14TFs iENDO early and late passage cells is normal by aCGH array. a) aCGH array on passage 4 14TFs iENDO cells b) aCGH array on passage 20, 14TFs iENDO cells.
(TIF)

S6 Fig iENDO cells grafted under the kidney capsule differentiate into hepatocyte like cells after 3 weeks. A-E) Representative Immunohistochemistry analysis of iENDO derived grafts for AFP, ALB, AAT, HNF4A and OCT4 with respective isotype control staining at 3 weeks after transplantation. Representative for N = 3 independent experiments. Scale bar 50μm.
(TIF)

S7 Fig iENDO cells grafted under the kidney capsule differentiate into hepatocyte like cells after 3 months. A-E) Immunohistochemistry analysis of iENDO derived grafts for AFP, ALB, AAT (Scale bar 100μm), HNF4α and OCT4 (Scale bar 50μm), with respective isotype control staining at 3 months after transplantation. Representative for N = 7 independent experiments.
(TIFF)

S8 Fig iENDO cells grafted under the kidney capsule differentiate into pancreatic cells and intestinal like cell types 3 months after transplantation. A-B) Immunohistochemistry analysis of iENDO derived grafts for intestional marker Villin-C and Lysozyme and pancreatic endoderm PDX1 with respective isotype control staining at 3 months after transplantation. Representative for N = 7 independent experiments. Scale bar 50μm.
(TIFF)

S9 Fig iENDO cells grafted under the kidney capsule differentiate into cells expressing PDX1 but not hepatoblast proteins in mouse 2, 3 months after transplantation. A-E) Immunohistochemistry analysis of iENDO derived grafts for AFP, ALB, AAT, HNF4α and PDX1, with their respective isotype control staining at 3 months after transplantation. Scale bar 50μm.
(TIF)

S10 Fig iENDO cells grafted under the kidney capsule differentiate into pancreatic endocrine and intestinal cell types 3 months after transplantation (mouse 2). A-E) Immunohistochemistry analysis of iENDO derived graft for PDX1, NGN3, SST, CHGA, LYSOZYME, VILLIN-C, OCT4, with their respective isotype control staining at 3 months after transplantation. Scale bar 50μm.
(TIF)

S11 Fig iENDO derived tumor grafts do not express mesodermal or ectodermal lineage marker. A) Cardiac marker NKX2.5 staining on iENDO derived grafts and hESC derived cardiomyocytes. B) Neuronal marker Tubulin-III staining on iENDO derived grafts expressing ALB and HNF4A, and hiPSC derived neurons. C-D) q-RT-PCR analysis for neuronal and cardiac marker in iENDO grafts in comparison with hESC cardiomyocytes and hiPSC neuronal progeny. Relative gene expression (to *PPIG*, log scale) analysis is represented. Error bars represents standard error of mean of three independent experiments.
(TIF)

S12 Fig Graft of iENDO cells under kidney capsule express hepatocyte, pancreatic, and gut transcripts (after 3 weeks and 3 months). A-B) Hepatocyte marker expression, C-D) pancreatic endoderm and endocrine marker expression, E) gut tube marker expression F) transgene expression in transplanted iENDO cells in kidney graft after 3 weeks (N = 3) and after 3 months (N = 9) with comparison with untransplanted kidney and iENDO day 0 or before transplantation (N = 3). Relative qRT-PCR gene expression relative to the *PPIG* housekeeping gene in log scale after 3 weeks and 3 months. Error bars represents standard error mean of three independent experiments. *p<0.05, **p<0.01 and ***p<0.001 determined by unpaired 2-tailed Student’s t-test. NS- not significant.
(TIFF)

S13 Fig Generation of hepatocyte-like cells from iENDO cells in 3D using protocol B. A) Protocol time-line for hepatocyte differentiation in 3D from iENDO cells with or without DMSO in protocol B. B) Morphology of iENDO differentiated organoids at day 16 with and without DMSO (N = 3). C) Relative gene expression (to *PPIG*, log scale) in day 16 iENDO-HLCs ± DMSO represented as a heat-map for hepatocyte markers compared with hESC-HLCs organoids (d30) and PHHs (N = 3). D-F) Immunostaining for AFP, ALB and AAT on day 16 iENDO organoids ± DMSO with their respective isotype control. Scale bar 25 μm (representative example of N = 3). All data represent mean of three independent experiments. *p<0.05, **p<0.01 and ***p<0.001 determined by unpaired 2-tailed Student’s t-test.
(TIF)

S14 Fig No expression of pancreatic genes in iENDO-HLC cells and No expression of liver genes in iENDO derived pancreatic endocrine cells. A-B) Pancreatic endocrine genes did not express in iENDO differentiated into HLCs method 1 and method 2, with comparison of iENDO differentiated into PEC day 12 and Day 30 (N = 3). C-D) Hepatic endoderm genes were not express in iENDO differentiated into pancreatic progeny, on day 12 and day 30 (N = 3). Relative qRT-PCR gene expression relative to the *PPIG* housekeeping gene in log scale. Error bars represents standard error mean of three independent experiments. *p<0.05, **p<0.01 and ***p<0.001 determined by unpaired 2-tailed Student’s t-test. NS- not significant.
(TIF)

S15 Fig Transduction efficiency of human MAPC. A) Histogram plots for different dilutions of PLVX-eGFP viral vector transduced hMAPCs. B) Summary table indicating the percentage of eGFP positive cells obtained by transduction of hMAPC with different dilutions of viral vector. Representative for 3 independent experiments. (Note: Red highlighted 300μl of unconcentrated virus can infect hMAPC at efficiency of 96.57%).
(TIF)

S1 Table List of primer sequences used for PCR amplification of cDNA for cloning into PLVX-IRES-HYG lentiviral vector. (PDF)

S2 Table List of qRT-PCR-primers used for transgene expression analysis (CDS-IRES) based. (PDF)

S3 Table List of qRT-PCR-primers used for total gene expression analysis (Exon-exon spanning primer). (PDF)

S4 Table List of qRT-PCR primers used for endogeneous gene expression analysis (CDS-3’UTR or 5’UTR-CDS). (PDF)

S5 Table List of qRT-PCR primers used for primitive endoderm, mesendoderm, hepatocytes, pancreatic endocrine cells (Exon-exon spanning primers or CDS-3’UTR or 5’UTR-CDS). (PDF)

S6 Table List of primary and secondary antibodies used for immunostaining and immunohistochemistry. (PDF)

S7 Table List of primary and secondary antibodies used for immunostaining and immunohistochemistry. (PDF)

S8 Table List of isotype antibodies used for immunostaining and immunohistochemistry. (PDF)

S9 Table List of FACS antibodies. (PDF)
