# Supplementary figures and images for: Generation of hepatocyte- and endocrine pancreatic-like cells from human induced endodermal progenitor cells
Source: PLoS One. 2018 May 11;13(5):e0197046. doi: 10.1371/journal.pone.0197046 (PMC5947914; doi:10.1371/journal.pone.0197046)

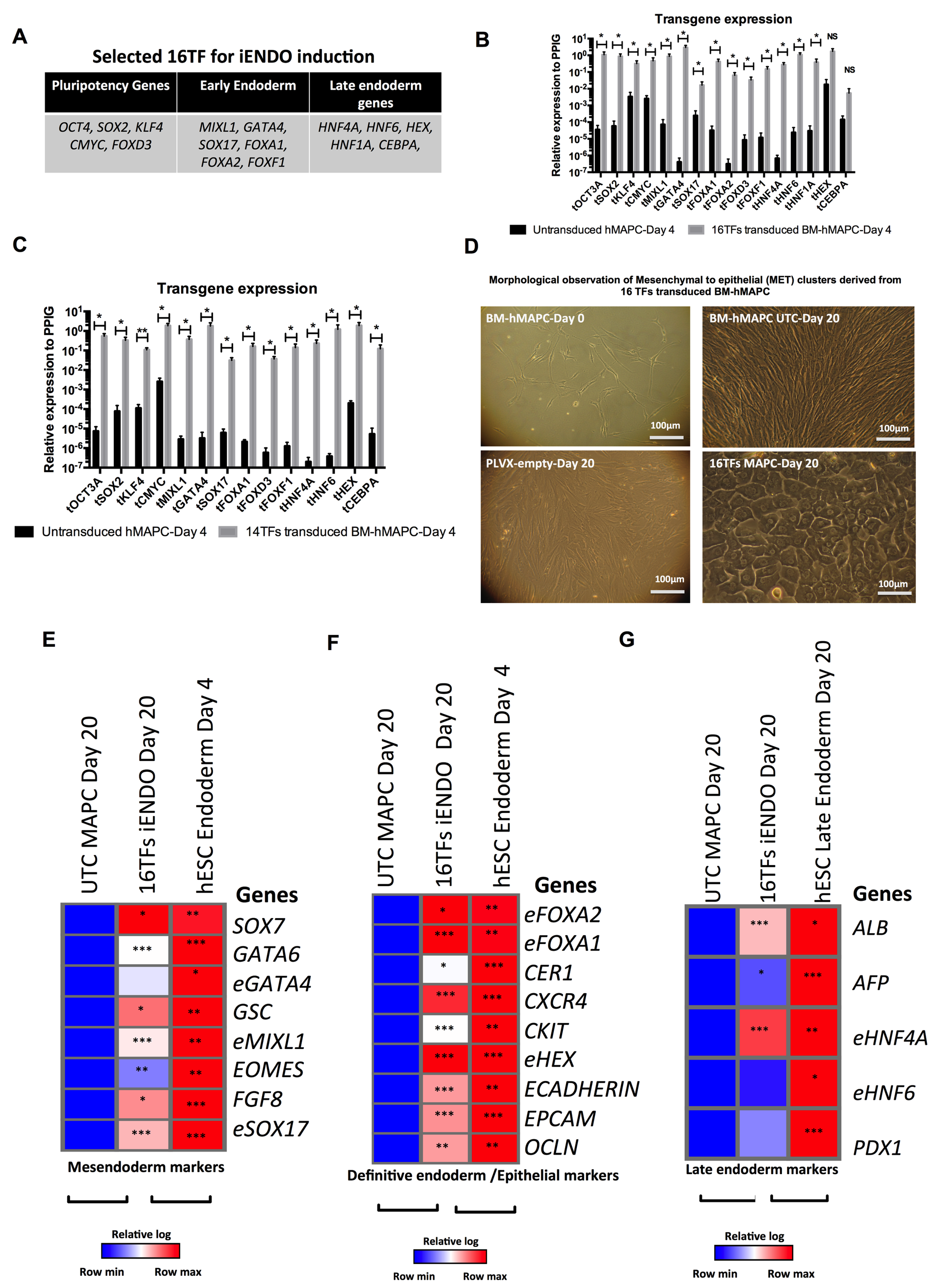

Supplement: S1 Fig — A) Selected list of 16 transcription factors. B) Transgene expression analysis by qRT-PCR after 16TF transduction on day 4 C) Transgene expression analysis by qRT-PCR after 14TF transduction on day 4. D) Morphological changes of 14TF transduced hMAPC from day 0 to day 20 after transduction. Untransduced BM-hMAPC and PLVX-eGFP transduced BM hMAPC at day 20 did not show morphological changes (scale bar 100μm). E-G) Relative gene expression (to PPIG, log scale) in day 20 16TF transduced cells represented as a heat-map for mesendoderm (E), definitive endoderm and epithelial marker genes (F) and late endoderm marker genes (G) compared with untransduced hMAPCs and hESC derived endodermal progenitors (day 4) or mature endodermal cells (day 20). All data represent mean of three independent experiments.*p<0.05, **p<0.01 and ***p<0.001 determined by unpaired 2-tailed Student’s t-test. (TIF) [file pone.0197046.s001.tif]

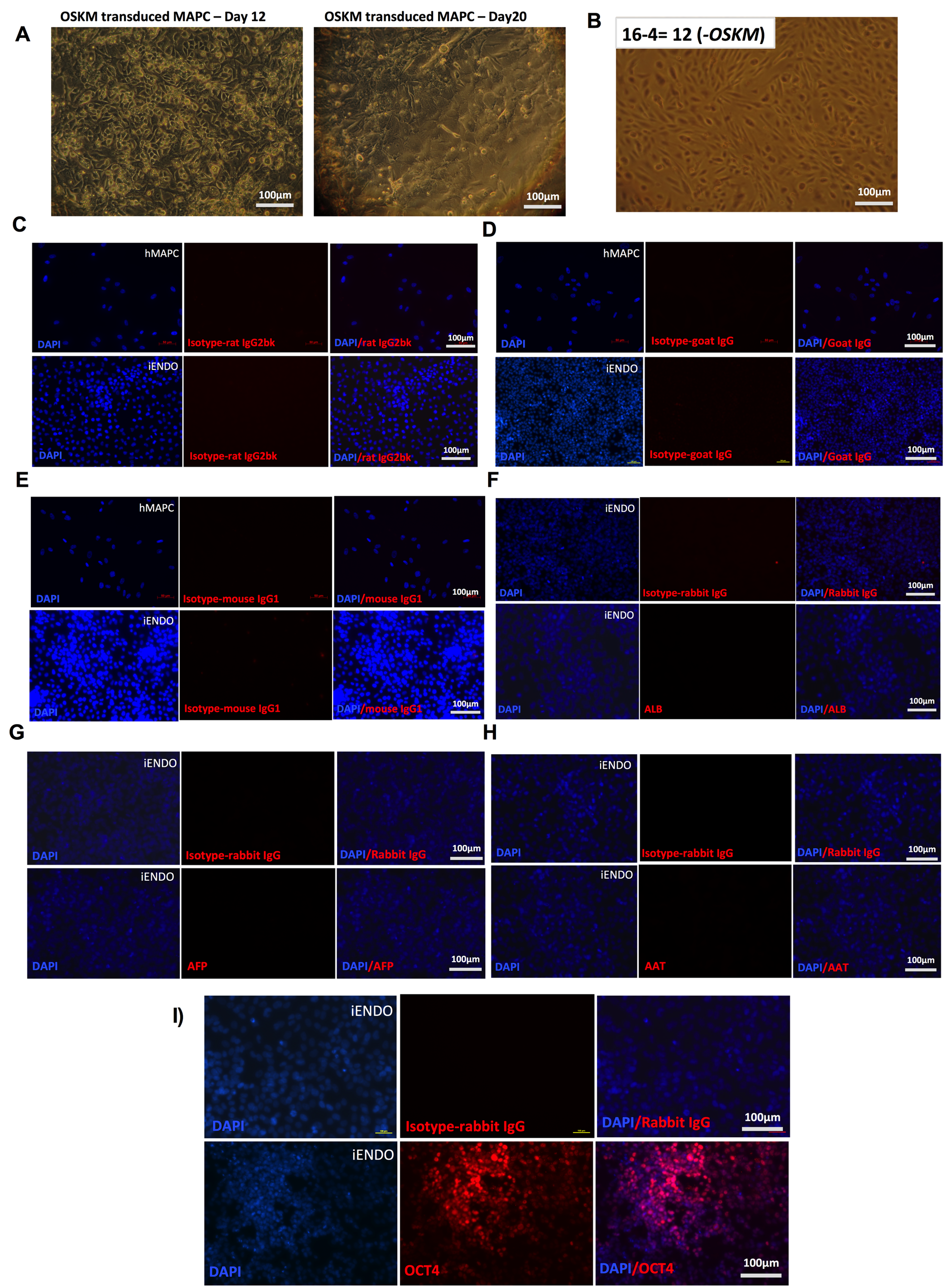

Supplement: S2 Fig — A) cuboidal morphology was seen when hMAPC cells were transduced with OSKM alone and endoderm induction medium from day 12 onwards. B) Cuboidal morphological changes were not observed in hMAPC transduced without OSKM (12TFs). C-E) Isotype control staining for CXCR4, SOX17 and CK 18 antibody in hMAPC and iENDO cells. F-H) 14TF iENDO stained for ALB, AFP and AAT with respective isotype controls. I) 14TF iENDO stained for OCT4 with respective isotype control. Representative for 3 independent experiments. Scale bar 100μm. (TIF) [file pone.0197046.s002.tif]

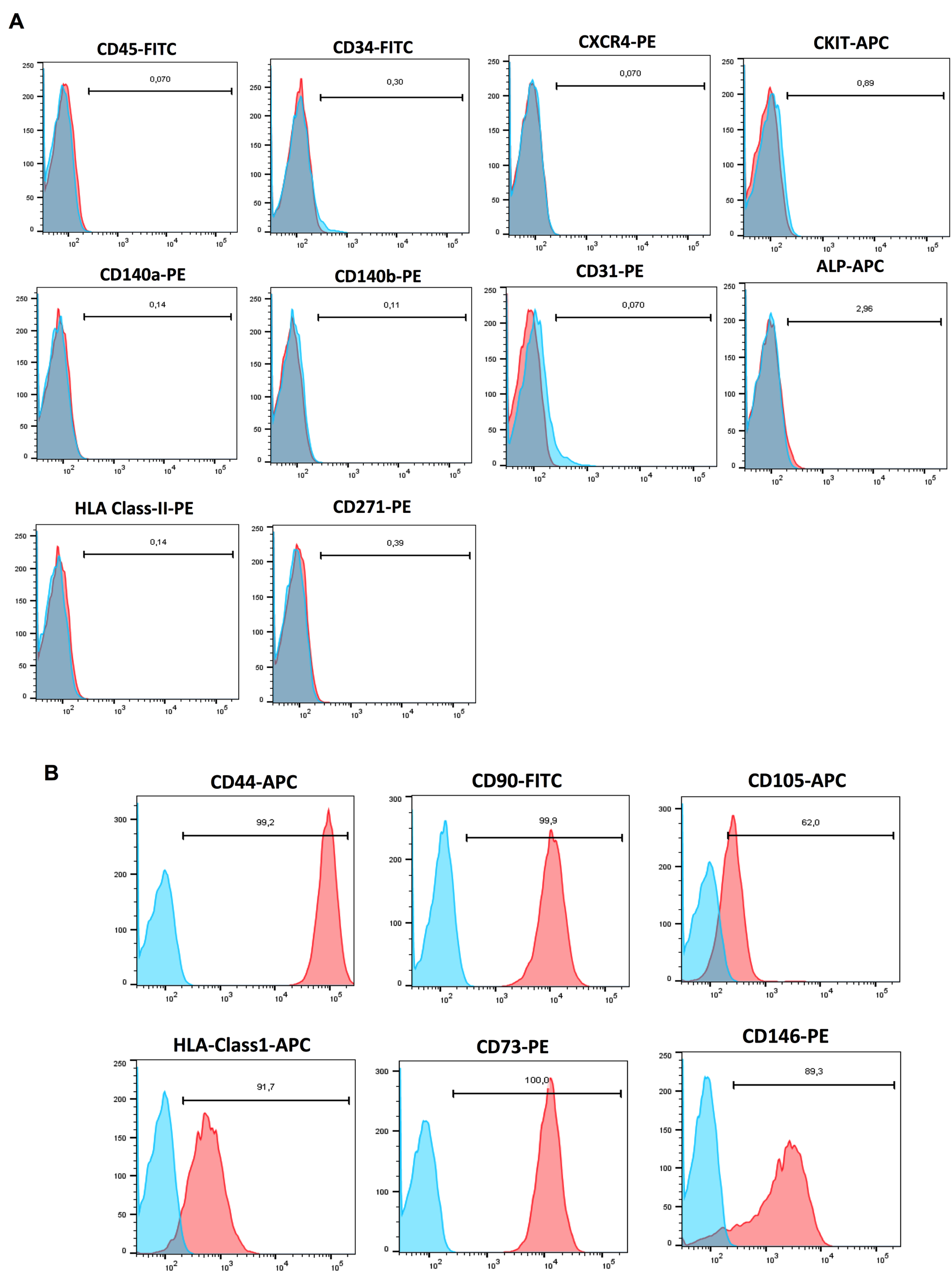

Supplement: S3 Fig — A) Histograms for surface markers: CD45-FITC, CD34-FITC, CXCR4-PE, CKIT-APC, CD140a-PE, CD140b-PE, CD31-PE, ALP-APC, HLA Class-II-PE, and CD271-PE with respective isotype control. B) Histogram for surface markers: CD44-APC, CD90-FITC, CD105-APC, HLA Class1-APC, CD73-PE, and CD146-PE with respective isotype control. Representative for 3 independent experiments. (TIF) [file pone.0197046.s003.tif]

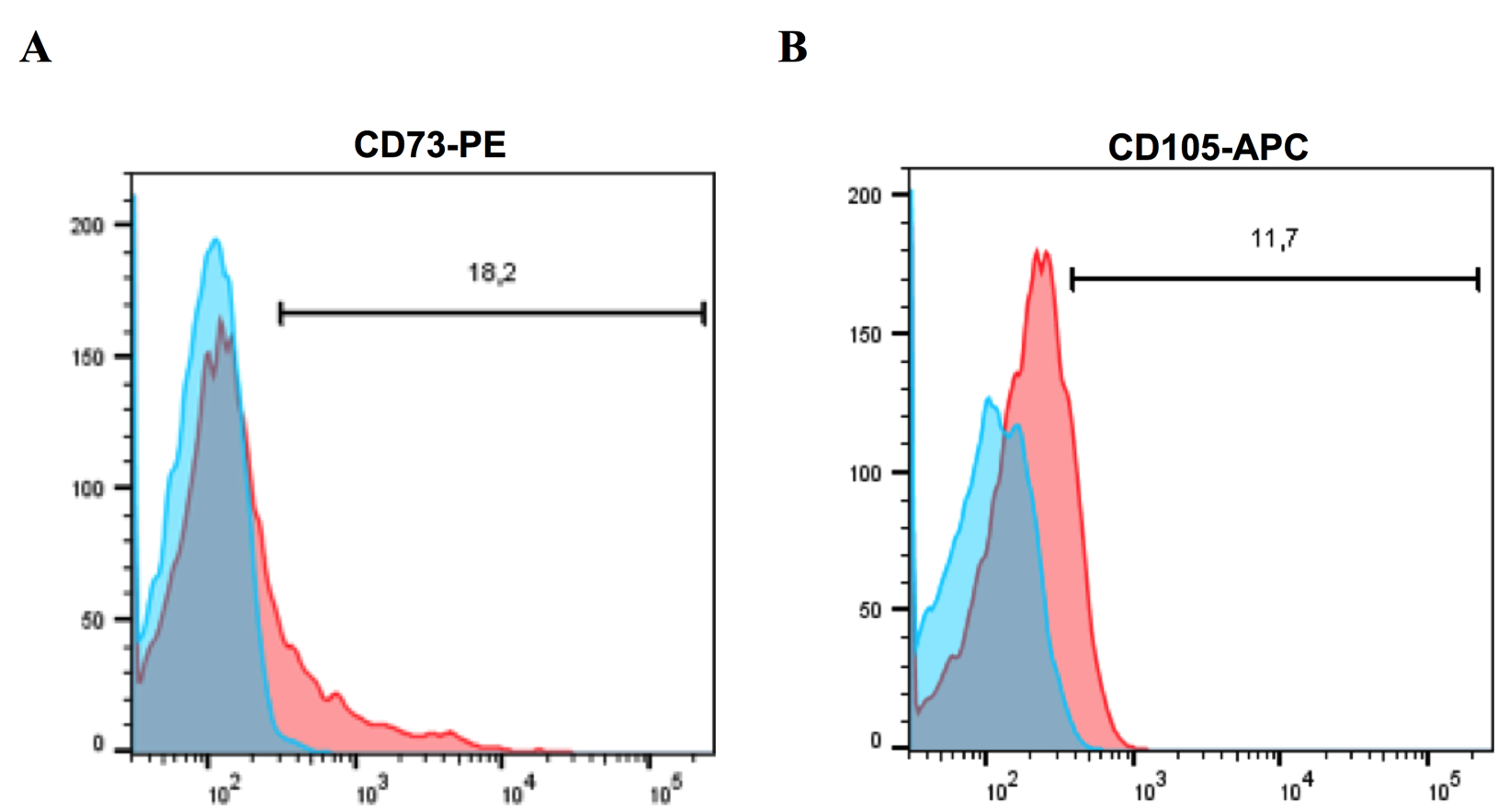

Supplement: S4 Fig — A-B) Histograms for hMAPC surface markers staining on 14TFs iENDO cells: CD73-PE and CD105-APC with respective isotype control. Representative for 3 independent experiments. (TIFF) [file pone.0197046.s004.tiff]

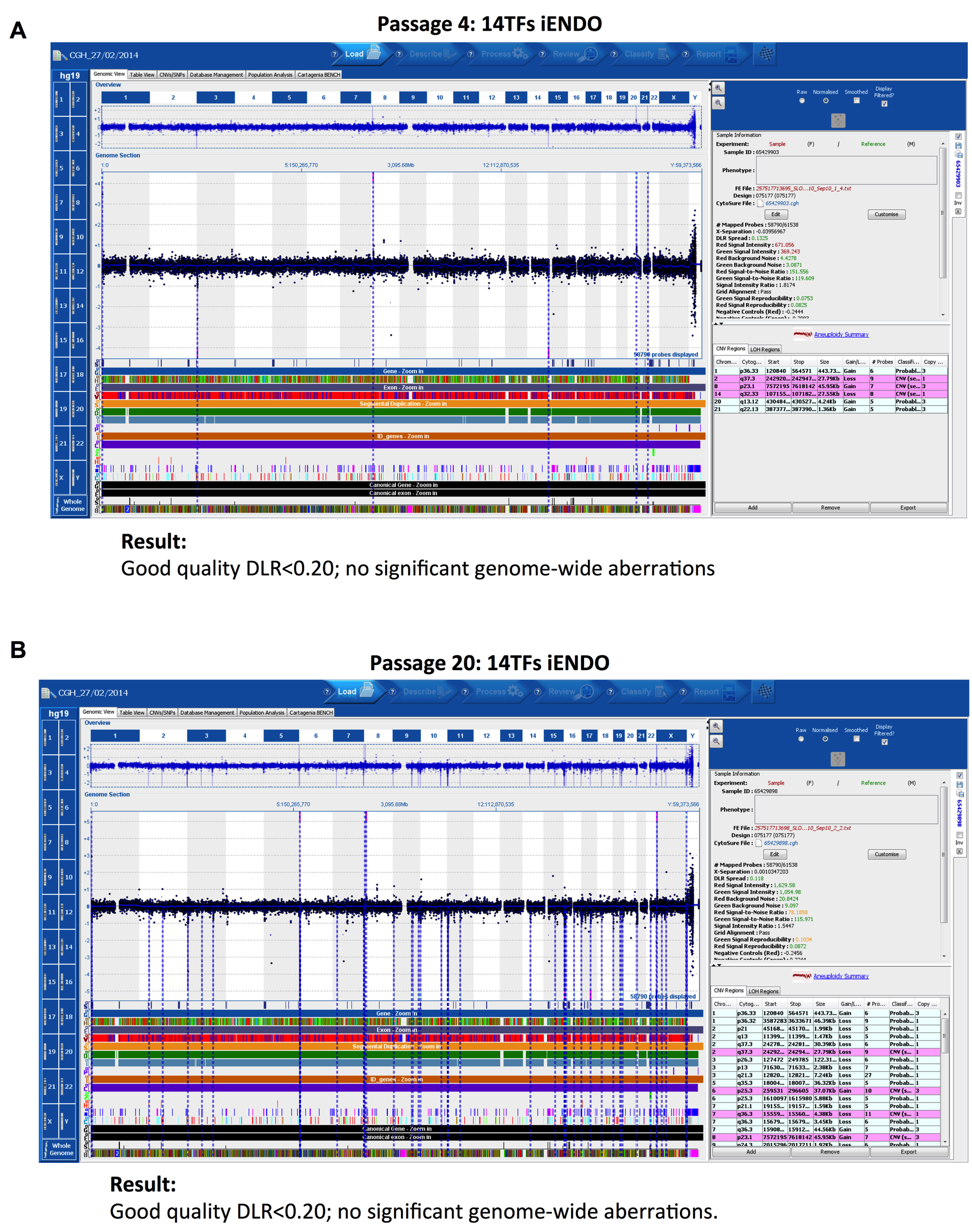

Supplement: S5 Fig — a) aCGH array on passage 4 14TFs iENDO cells b) aCGH array on passage 20, 14TFs iENDO cells. (TIF) [file pone.0197046.s005.tif]

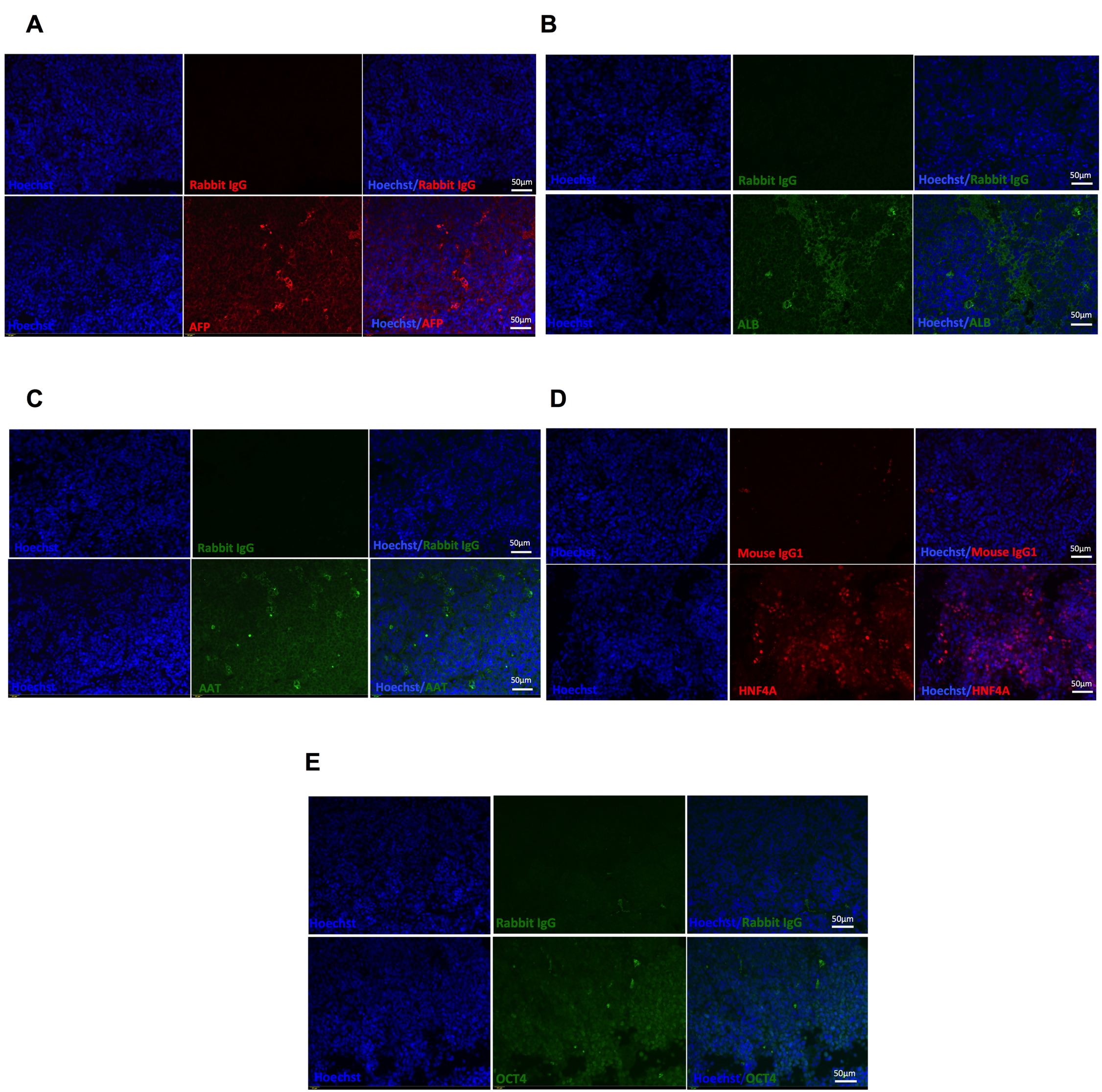

Supplement: S6 Fig — A-E) Representative Immunohistochemistry analysis of iENDO derived grafts for AFP, ALB, AAT, HNF4A and OCT4 with respective isotype control staining at 3 weeks after transplantation. Representative for N = 3 independent experiments. Scale bar 50μm. (TIF) [file pone.0197046.s006.tif]

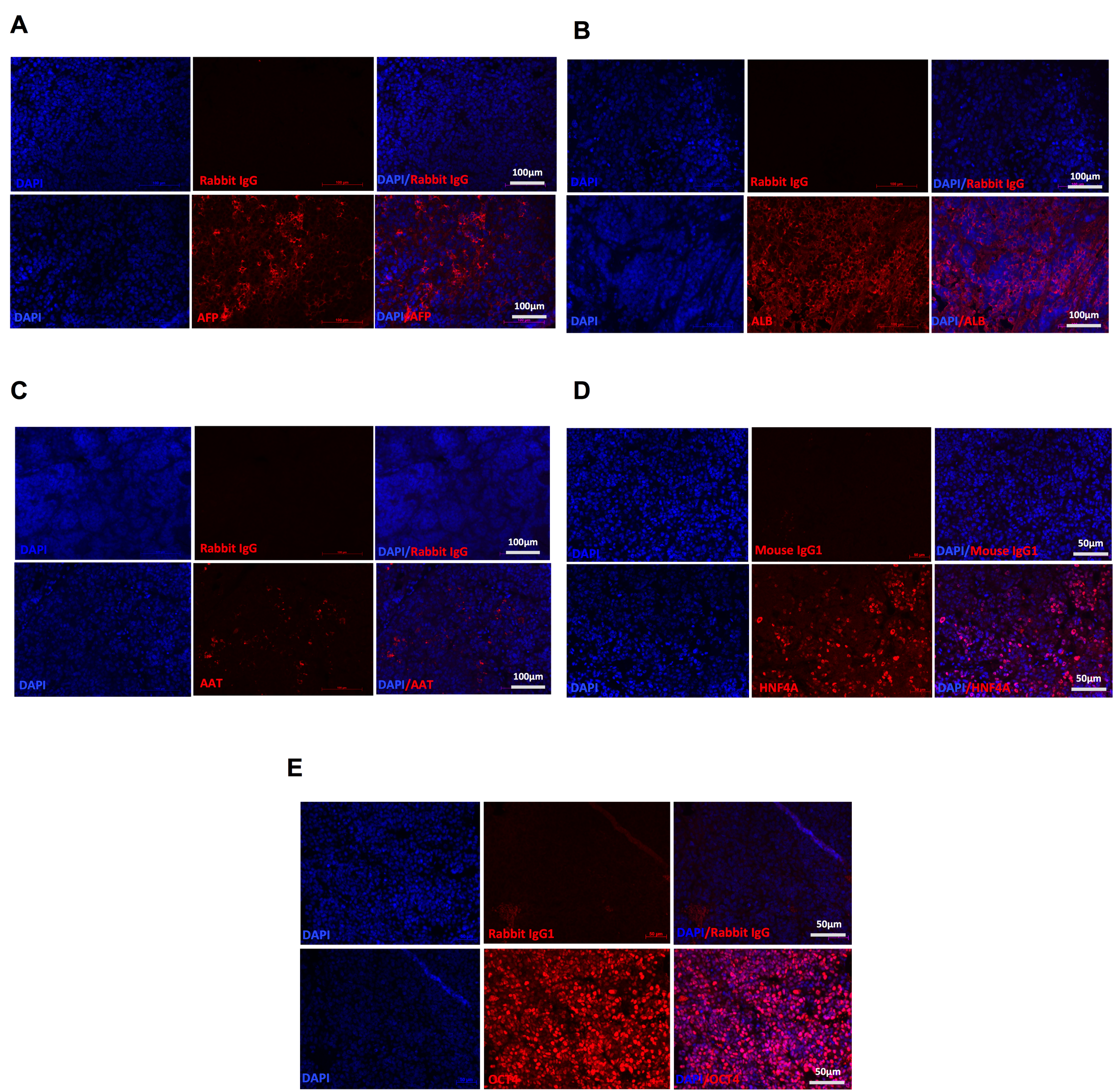

Supplement: S7 Fig — A-E) Immunohistochemistry analysis of iENDO derived grafts for AFP, ALB, AAT (Scale bar 100μm), HNF4α and OCT4 (Scale bar 50μm), with respective isotype control staining at 3 months after transplantation. Representative for N = 7 independent experiments. (TIFF) [file pone.0197046.s007.tiff]

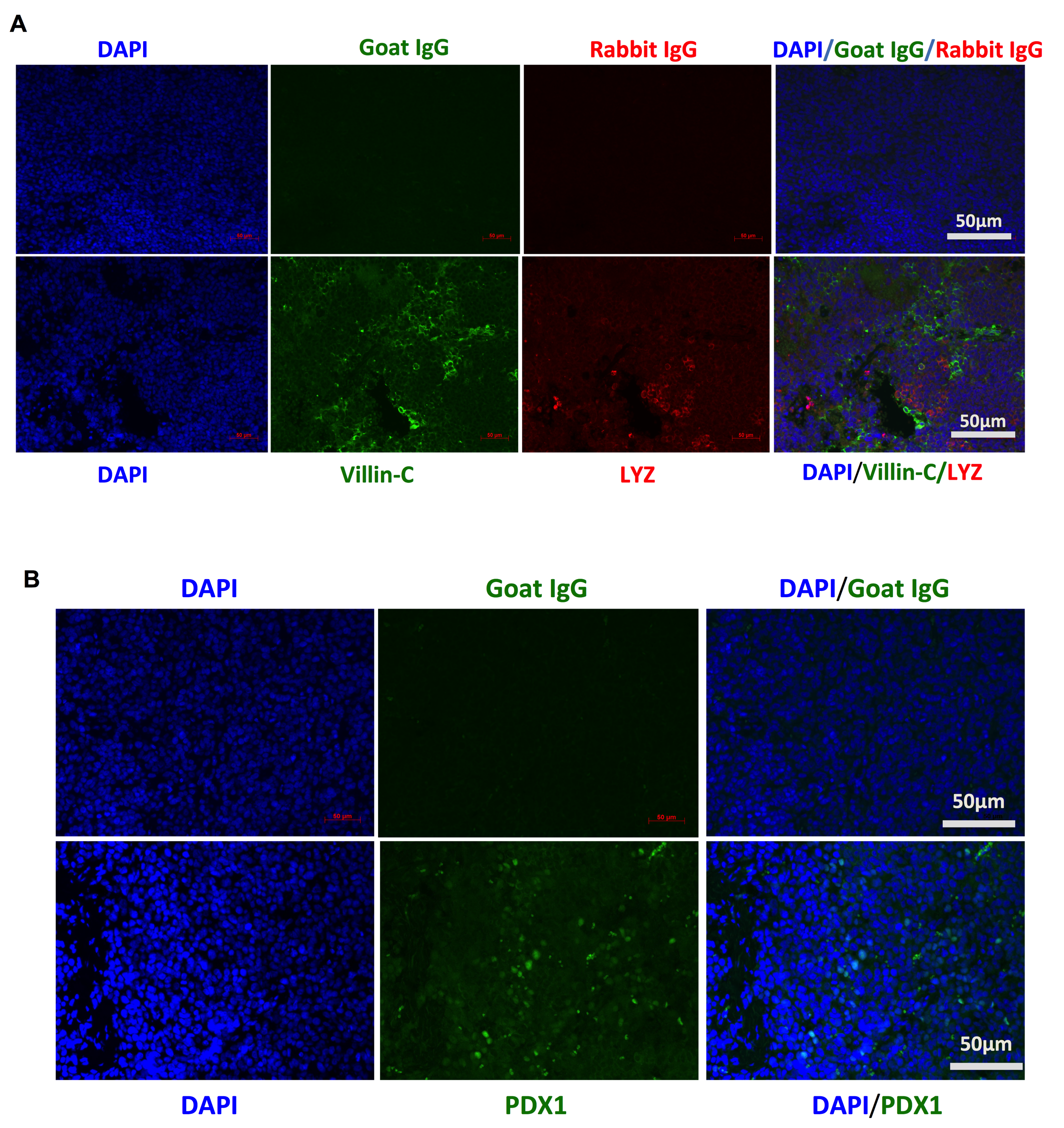

Supplement: S8 Fig — A-B) Immunohistochemistry analysis of iENDO derived grafts for intestional marker Villin-C and Lysozyme and pancreatic endoderm PDX1 with respective isotype control staining at 3 months after transplantation. Representative for N = 7 independent experiments. Scale bar 50μm. (TIFF) [file pone.0197046.s008.tiff]

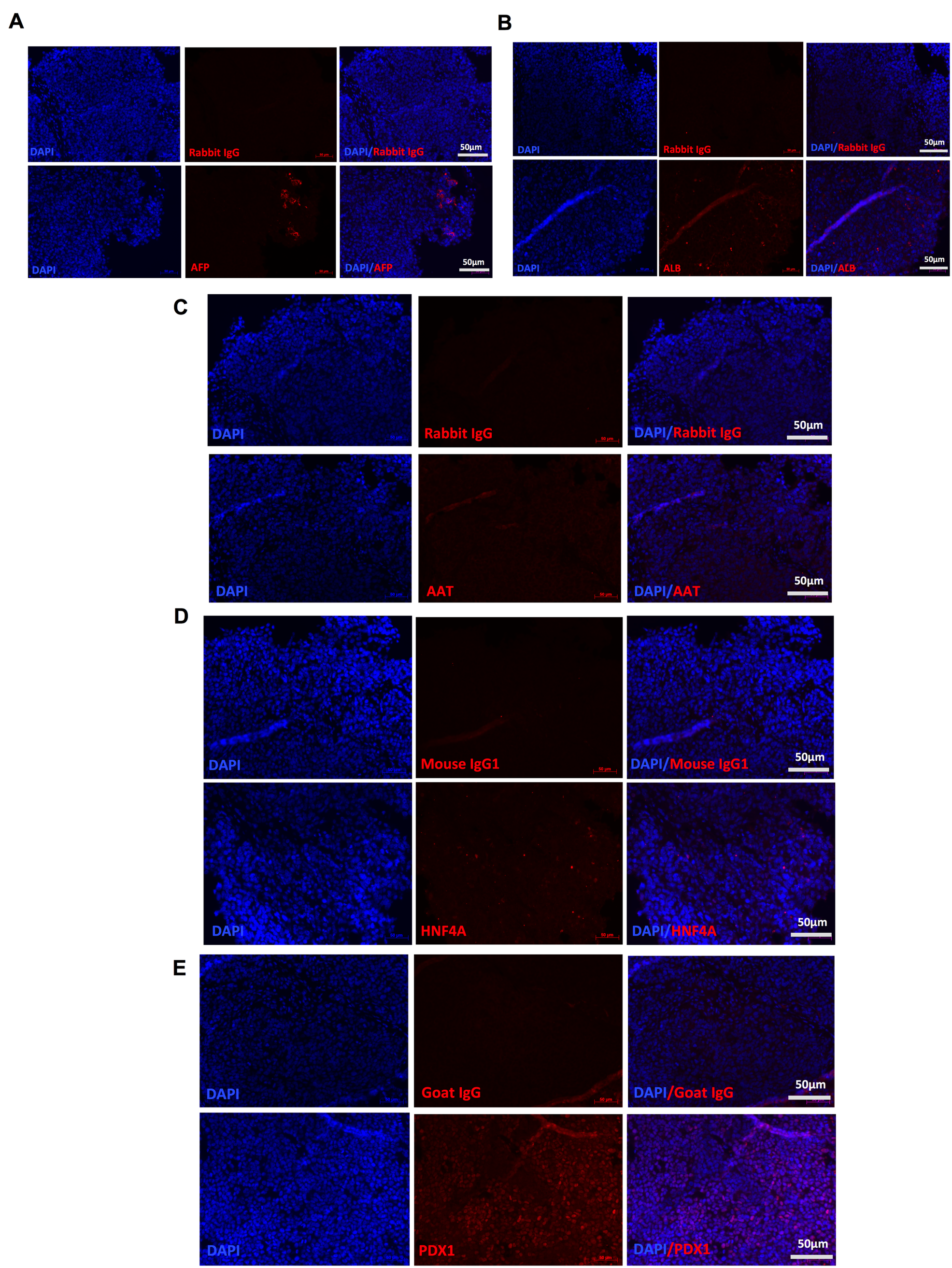

Supplement: S9 Fig — A-E) Immunohistochemistry analysis of iENDO derived grafts for AFP, ALB, AAT, HNF4α and PDX1, with their respective isotype control staining at 3 months after transplantation. Scale bar 50μm. (TIF) [file pone.0197046.s009.tif]

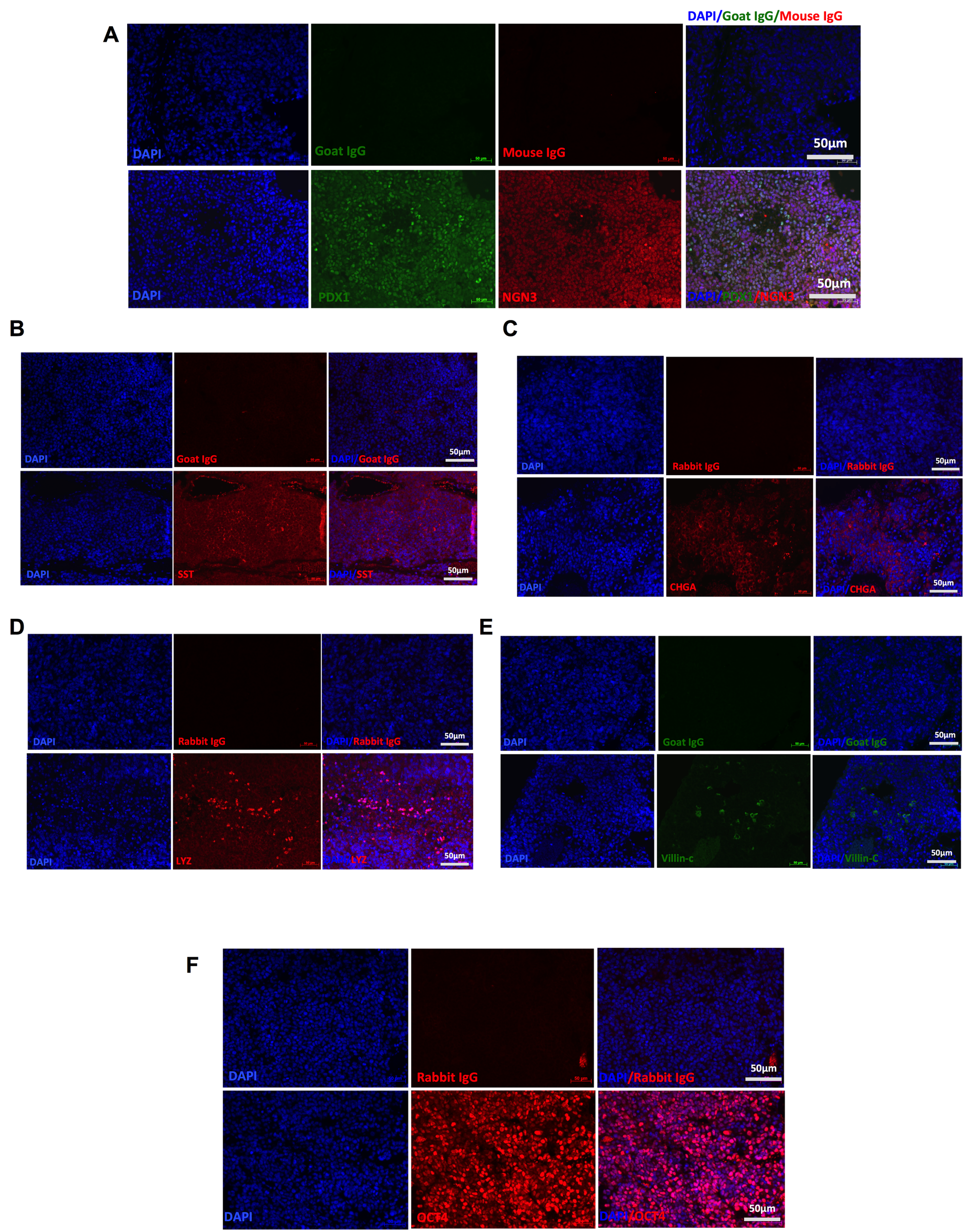

Supplement: S10 Fig — A-E) Immunohistochemistry analysis of iENDO derived graft for PDX1, NGN3, SST, CHGA, LYSOZYME, VILLIN-C, OCT4, with their respective isotype control staining at 3 months after transplantation. Scale bar 50μm. (TIF) [file pone.0197046.s010.tif]

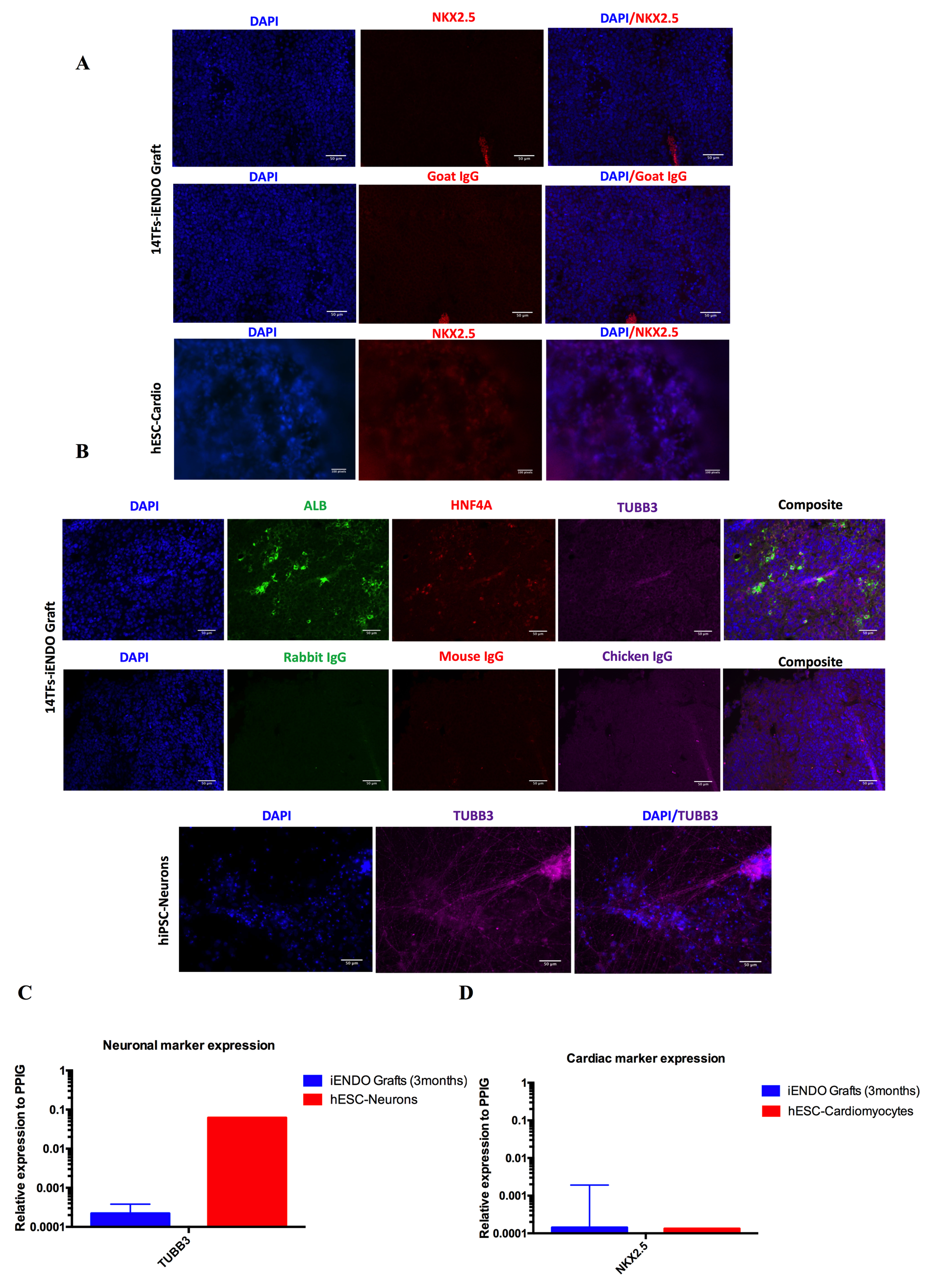

Supplement: S11 Fig — A) Cardiac marker NKX2.5 staining on iENDO derived grafts and hESC derived cardiomyocytes. B) Neuronal marker Tubulin-III staining on iENDO derived grafts expressing ALB and HNF4A, and hiPSC derived neurons. C-D) q-RT-PCR analysis for neuronal and cardiac marker in iENDO grafts in comparison with hESC cardiomyocytes and hiPSC neuronal progeny. Relative gene expression (to PPIG, log scale) analysis is represented. Error bars represents standard error of mean of three independent experiments. (TIF) [file pone.0197046.s011.tif]

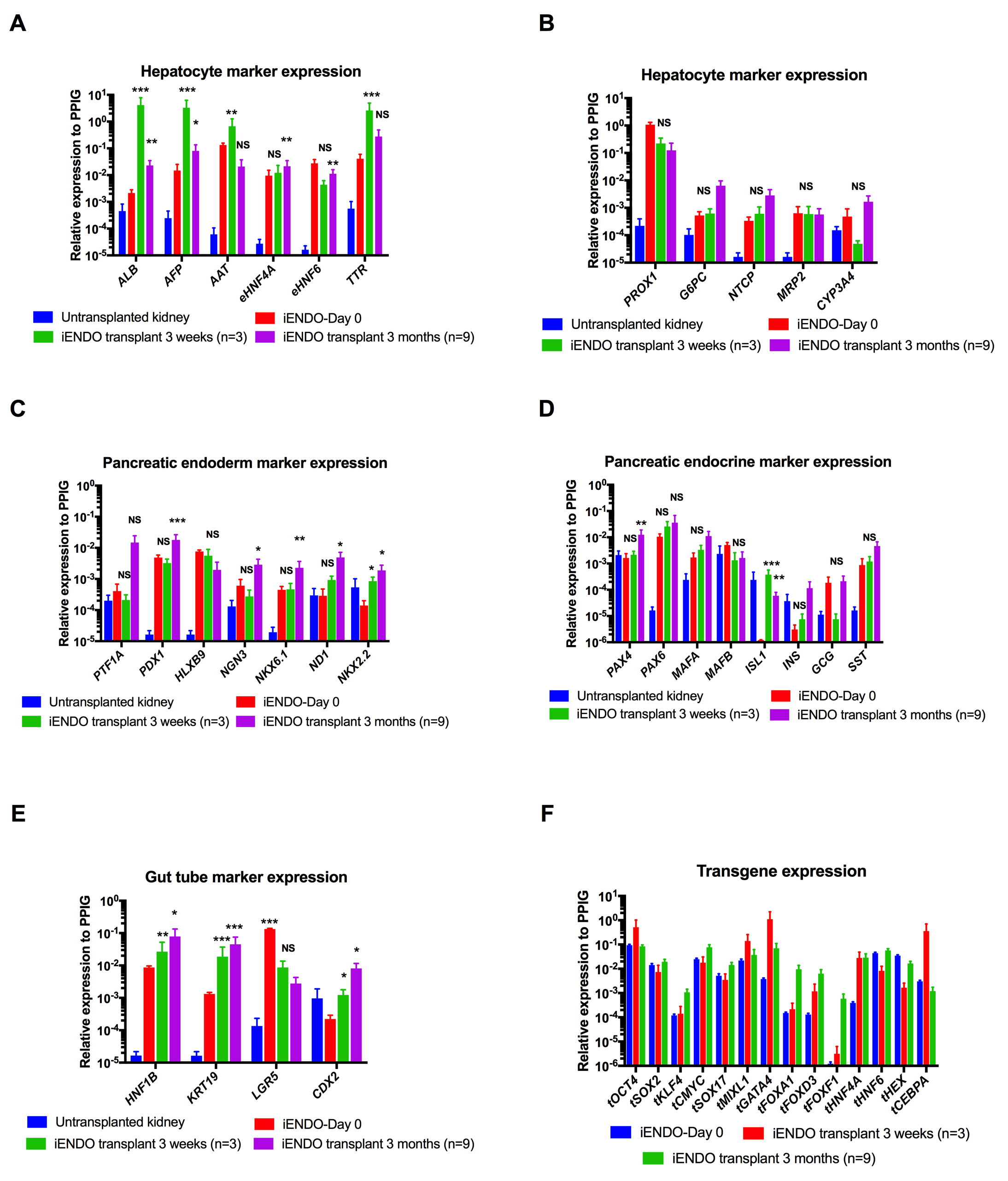

Supplement: S12 Fig — A-B) Hepatocyte marker expression, C-D) pancreatic endoderm and endocrine marker expression, E) gut tube marker expression F) transgene expression in transplanted iENDO cells in kidney graft after 3 weeks (N = 3) and after 3 months (N = 9) with comparison with untransplanted kidney and iENDO day 0 or before transplantation (N = 3). Relative qRT-PCR gene expression relative to the PPIG housekeeping gene in log scale after 3 weeks and 3 months. Error bars represents standard error mean of three independent experiments. *p<0.05, **p<0.01 and ***p<0.001 determined by unpaired 2-tailed Student’s t-test. NS- not significant. (TIFF) [file pone.0197046.s012.tiff]

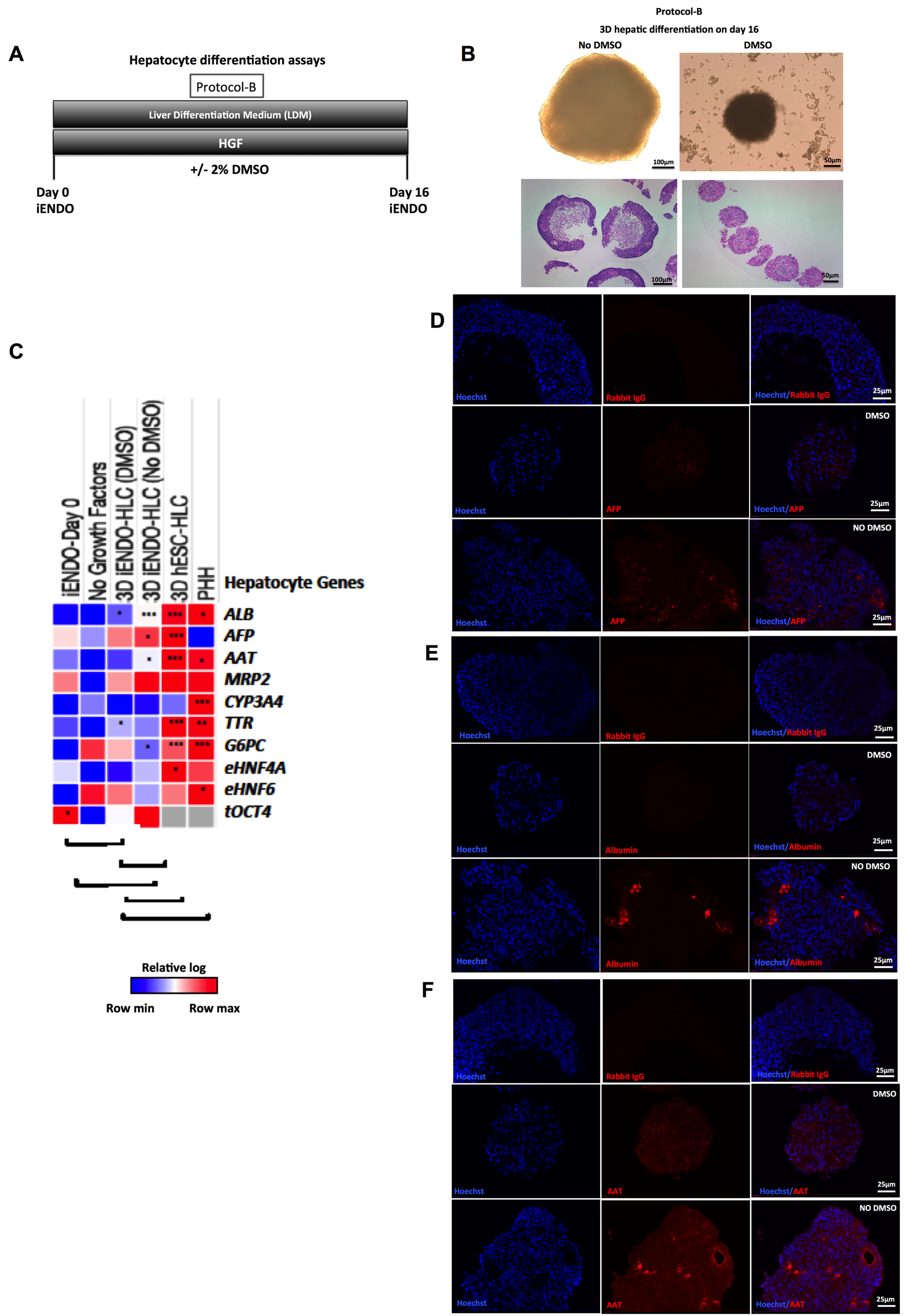

Supplement: S13 Fig — A) Protocol time-line for hepatocyte differentiation in 3D from iENDO cells with or without DMSO in protocol B. B) Morphology of iENDO differentiated organoids at day 16 with and without DMSO (N = 3). C) Relative gene expression (to PPIG, log scale) in day 16 iENDO-HLCs ± DMSO represented as a heat-map for hepatocyte markers compared with hESC-HLCs organoids (d30) and PHHs (N = 3). D-F) Immunostaining for AFP, ALB and AAT on day 16 iENDO organoids ± DMSO with their respective isotype control. Scale bar 25 μm (representative example of N = 3). All data represent mean of three independent experiments. *p<0.05, **p<0.01 and ***p<0.001 determined by unpaired 2-tailed Student’s t-test. (TIF) [file pone.0197046.s013.tif]

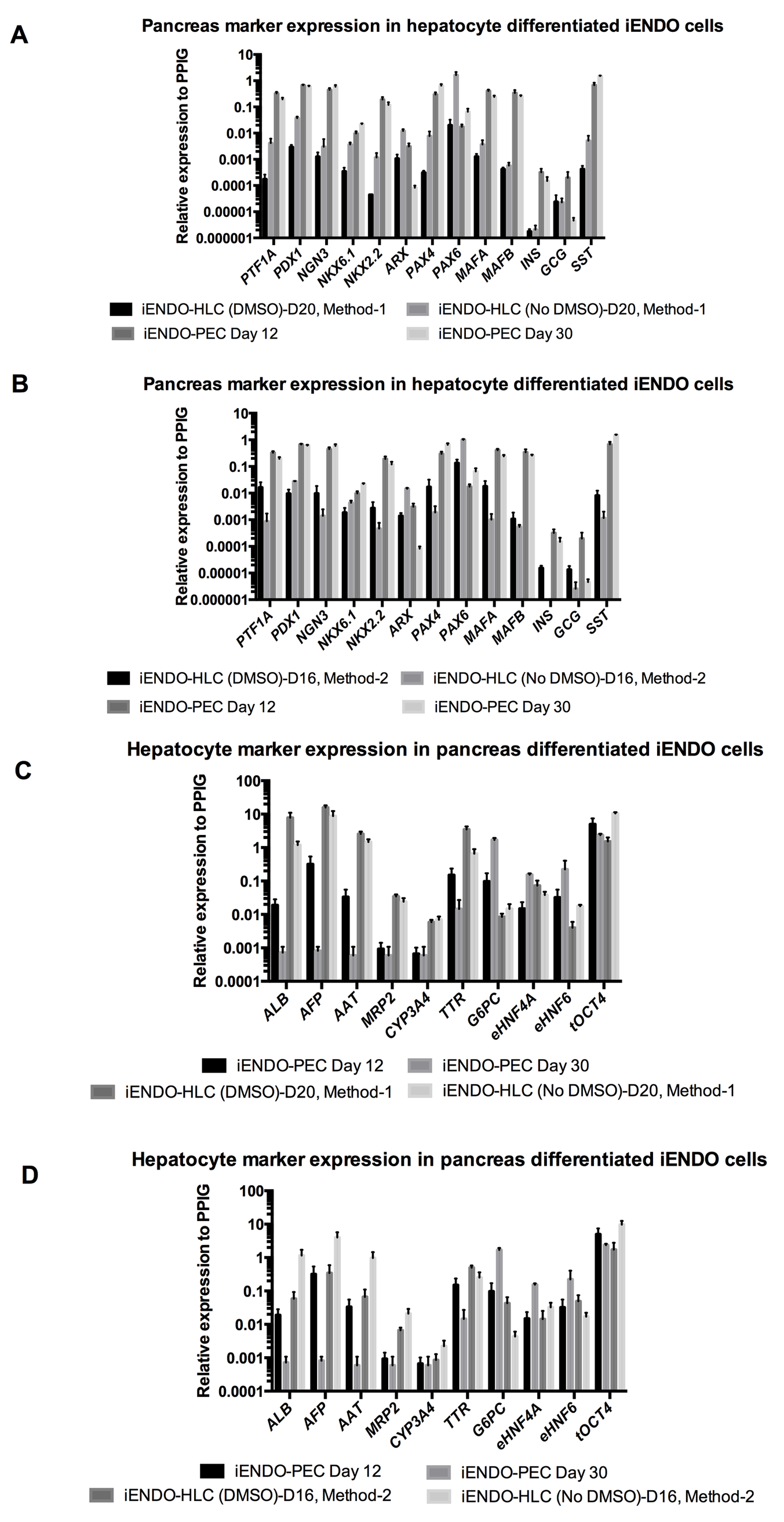

Supplement: S14 Fig — A-B) Pancreatic endocrine genes did not express in iENDO differentiated into HLCs method 1 and method 2, with comparison of iENDO differentiated into PEC day 12 and Day 30 (N = 3). C-D) Hepatic endoderm genes were not express in iENDO differentiated into pancreatic progeny, on day 12 and day 30 (N = 3). Relative qRT-PCR gene expression relative to the PPIG housekeeping gene in log scale. Error bars represents standard error mean of three independent experiments. *p<0.05, **p<0.01 and ***p<0.001 determined by unpaired 2-tailed Student’s t-test. NS- not significant. (TIF) [file pone.0197046.s014.tif]

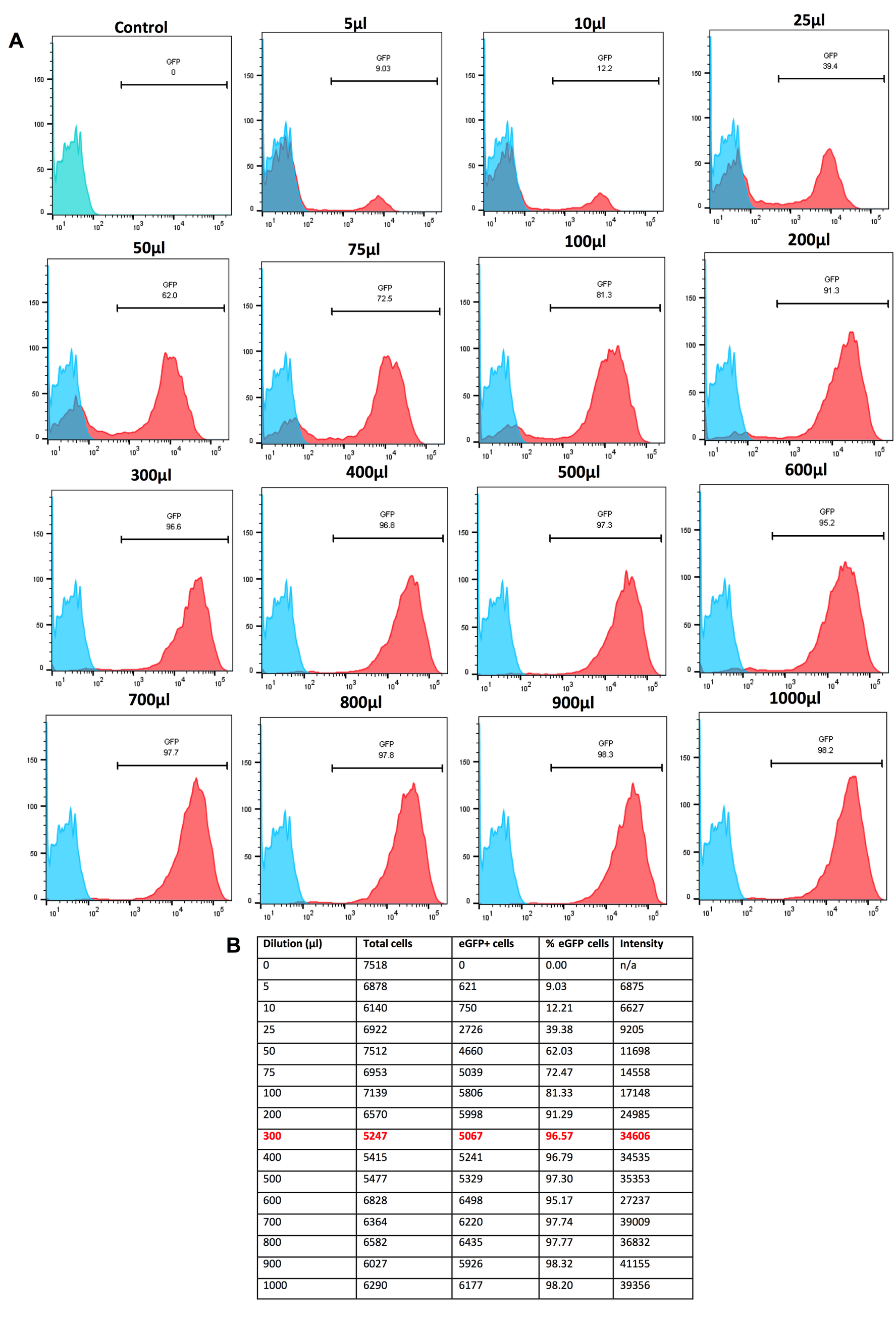

Supplement: S15 Fig — A) Histogram plots for different dilutions of PLVX-eGFP viral vector transduced hMAPCs. B) Summary table indicating the percentage of eGFP positive cells obtained by transduction of hMAPC with different dilutions of viral vector. Representative for 3 independent experiments. (Note: Red highlighted 300μl of unconcentrated virus can infect hMAPC at efficiency of 96.57%). (TIF) [file pone.0197046.s015.tif]
