# Supplementary material for: Generation of hepatocyte- and endocrine pancreatic-like cells from human induced endodermal progenitor cells
Source: PLoS One. 2018 May 11;13(5):e0197046. doi: 10.1371/journal.pone.0197046 (PMC5947914; doi:10.1371/journal.pone.0197046)
Supplement: S1 Table — (PDF) [file pone.0197046.s016.pdf]

S1 Table. List of primer sequences used for PCR amplification of cDNA for cloning into PLVX-IRES-HYG lentiviral vector

Genes	Primers (5' to 3' direction)	Res.sites	Type
OCT3A	AGTCTTCTAGAATGGCGGGACACCTGGCTTCG	Xba1	Forward
	ATGTAGGATCCTCAGTTTGAATGCATGGGAG	BamH1	Reverse
SOX2	ATCGTCCTCGAGATGTACAACATGATGGAG	Xho1	Forward
	ATGTATCTAGATCACATGTGTGAGAGGGGCAG	Xba1	Reverse
KLF4	ATCGTCCTCGAGATGAGGCAGCCACCTG	Xho1	Forward
	ATGTATCTAGATTAAAAATGCCTCTTCATGTGTAA	Xba1	Reverse
CMYC	ATCGTCCTCGAGCTGGATTTTTTTCGG	Xho1	Forward
	ATGTATCTAGATTACGCACAAGAGTTCCGTA	Xba1	Reverse
MIXL1	ATCGTCCTCGAGCCACCATGGCCACAGCCGAGTC	Xho1	Forward
	TAGTATCTAGATCAAAAGTTACCAAAGGCAGAAAAGATG	Xba1	Reverse
GATA4	AGTCTCTCGAGGCCACCATGTATCAGAGCTTGGCCATG	XhoI	Forward
	TAGTATCTAGATTACGCAGTGATTATGTCCC	Xba I	Reverse
SOX17	ATCGTCCTCGAGCACCATGAGCAGCCCGGATGCGGG	Xho1	Forward
	ATGACTCTAGATCACACGTCAGGATAGTTGCAGT	Xba1	Reverse
FOXA1	ATCGTCCTCGAGGCCACCATGTTAGGAACTGTGAAGATGG	XhoI	Forward
	TAGACTCTAGACTAGGAAGTGTTTAGGACG	Xba I	Reverse
FOXA2	ATCGACTCTAGAATGCACTCGGCTTCCAGTATGCTG	Xba1	Forward
	ATGACGGATCCGCCGTCGTCTTCTTAAGAGGAG	BamH1	Reverse
FOXD3	AGTCTCTCGAGGCCACCATGACCCTCTCCGGCGGCAG	Xho1	Forward
	TAGTATCTAGACTATTGCGCCGGCCATTTGGCTT	Xba1	Reverse
FOXF1	AGTCTCTCGAGGCCACCATGTCTTCGGCGCCCGAGAAGC	Xho1	Forward
	TAGTATCTAGATCACATCACGCAAGGCTTGATGTCTTGGTAG	Xba1	Reverse
HNF4α	AGTCTTCTAGAGCCACCATGCGACTCTCCAAAACCCTCG	Xba1	Forward
	TAGTAGCGGCCGCCTAGATAACTTCCTGCTTGGTGATGGTCG	Not1	Reverse
HNF6	ATCGTCCTCGAGGCCACCATGAACGCGCAGCTGAC	Xho1	Forward
	TAGTATCTAGATCATGCTTTGGTACAAGTGCT	Xba1	Reverse
HNF1α	AGTCTTCTAGAGCCACCATGGTTTCTAAACTGAGCCA	Xba1	Forward
	ATGTAGGATCCTTACTGGGAGGAAGAGGC	BamH1	Reverse
HEX	ATCGTCCTCGAGCACCATGCAGTACCCGCACCCCGG	Xho1	Forward
	ATGACTCTAGATCATCCAGCATTAAAATAGCTTTTATC	Xba1	Reverse
CEBPα	AGTCTACTAGTGCCACCATGGAGTCGGCCGACTTCTACG	Spe1	Forward
	ATGTAGGATCCTCACGCGCAGTTGCCCATG	BamH1	Reverse

* The underlined sequence denotes the sites for restriction enzymes.
