# Supplementary material for: Generation of hepatocyte- and endocrine pancreatic-like cells from human induced endodermal progenitor cells
Source: PLoS One. 2018 May 11;13(5):e0197046. doi: 10.1371/journal.pone.0197046 (PMC5947914; doi:10.1371/journal.pone.0197046)
Supplement: S2 Table — (PDF) [file pone.0197046.s017.pdf]

S2 Table. List of qRT-PCR primers used for transgene expression analysis (CDS-IRES) based.

Genes	Primer sequence
tOCT3A-F	CCATTTTGGTACCCCAGGCT
tSOX2-F	CCCCAGCAGACTTCACATGT
tKLF4-F	GCTGTGGATGGAAATTCGCC
tcMyc-F	GCATACATCCTGTCCGTCCA
tMIXL1-F	GTAGATGTGAACTGCCTGCC
tGATA4-F	CCTCTCGGCCCTGAAGCTCT
tSOX17-F	CGTGTGCAAGCCTGAGATGG
tFOXA1-F	CTGCCTCTAGGCAGCGCCTC
tFOXA2-F	ACAGGTGATGCACTACCCCG
tFOXD3-F	TCGCGCCCATTCTTAGCGTG
tFOXF1-F	CCTACTACCACCAGCAGGTC
tHNF4α-F	CAGGGTCTGAGCCCTATAAG
tHNF6-F	GCTGGGGTTGGAGCTGAGCA
tHNF1α-F	GTGTCCTCCAGCAGCCTGGT
tHEX-F	GCTCTCAATGTTCGCCCTCC
tCEBPα-F	TGCTGGAGCTGACCAGTGAC
IRES-R	GCCTTATTCCAAGCGGCTTC

* The t denotes the transgenic. F-forward, R-reverse
