# Supplementary material for: Generation of hepatocyte- and endocrine pancreatic-like cells from human induced endodermal progenitor cells
Source: PLoS One. 2018 May 11;13(5):e0197046. doi: 10.1371/journal.pone.0197046 (PMC5947914; doi:10.1371/journal.pone.0197046)
Supplement: S3 Table — (PDF) [file pone.0197046.s018.pdf]

S3 Table. List of qRT-PCR primers used for total gene expression analysis (Exon-exon spanning primer)

Genes	Forward primer sequence	Reverse primer sequence
OCT3A	GATGGCGTACTGTGGGCCC	TGGGACTCCTCCGGGTTTTG
SOX2	GAGTGGAAACTTTTGTCGGAGA	AGCGTGTACTTATCCTTCTTCAT
KLF4	GGTGCCCCGAATAACCGCTG	CTCTCTCCGAGGTAGGGGCG
CMYC	TGCTCCATGAGGAGACACC	CTCTGACCTTTTGCCAGGAG
MIXL1	GGATCCAGGTATGGTTCCAG	CATGAGTCCAGCTTTGAACC
GATA4	TCCAAACCAGAAAACGGAAG	CTGTGCCCGTAGTGAGATGA
SOX17	CGCTTTCATGGTGTGGGCTAAGGACG	TAGTTGGGGTGGTCCTGCATGTGCTG
FOXA1	AGGCCTACTCCTCCGTCCCG	CTAGGCCCGGGTTGGCATAGG
FOXA2	GCACTCGGCTTCCAGTATGC	CTCATGTACGTGTTCATGCCGT
FOXD3	AGTCGGCAGCGCTCATGGCC	CTATTGCGCCGGCCATTTGGC
FOXF1	CGGTATCACTCGCAGTCGCC	TCACATCACGCAAGGCTTGATGT
HNF4α	ACTACGGTGCCTCGAGCTGT	GGCACTGGTTCCTCTTGTCT
HNF6	AAATCACCATTTCCCAGCAG	ACTCCTCCTTCTTGCGTTCA
HNF1α	ACACCTCAACAAGGGCACTC	TGGTAGCTCATCACCTGTGG
HEX	CCCCCTGGGCAAACCTCTACT	GCCAGACGCTTCCTCTCGG
CEBPα	TCGGCCGACTTCTACGAGGC	GATGGACGTCTCGTGCTCGC
