# Supplementary material for: Generation of hepatocyte- and endocrine pancreatic-like cells from human induced endodermal progenitor cells
Source: PLoS One. 2018 May 11;13(5):e0197046. doi: 10.1371/journal.pone.0197046 (PMC5947914; doi:10.1371/journal.pone.0197046)
Supplement: S4 Table — (PDF) [file pone.0197046.s019.pdf]

S4 Table. List of qRT-PCR primers used for endogeneous gene expression analysis (CDS-3'UTR or 5'UTR-CDS).

Genes	Forward primer sequence	Reverse primer sequence
EOCT3A	GACAGGGGGAGGGGAGGAGCTAGG	CTTCCCTCCAACCAGTTGCCCCAAAC
ESOX2	TGGCGAACCATCTCTGTGGT	CCAACGGTGTCAACCTGCAT
EKLF4	ACGATCGTGGCCCCGGAAAAGGACC	TGATTGTAGTGCTTTCTGGCTGGGCTCC
ECMYC	GCATACATCCTGTCCGTCCA	TAGGTGATTGCTCAGGACAT
EMIXL1	GTAGATGTGAACTGCCTGCC	CATGGCTCCTCAGAGCTTAT
EGATA4	CCTCTCGGCCCTGAAGCTCT	CAAGTCCCAGGTCCGTGCAG
ESOX17	CGTGTGCAAGCCTGAGATGG	CTGCTTCTGGCCTGCAGGCT
EFOXA1	CTGCCTCTAGGCAGCGCCTC	CTTGCTACCAGCATGGCTAT
EFOXA2	AGGAGGAAAACGGGAAAGAA	GGTGCTTGAAGAAGCAGGAG
EFOXD3	AGTCGGCAGCGCTCATGGCC	TCCCGGCCATTGGCGCGTCC
EFOXF1	CGGTATCACTCGCAGTCGCC	CCGCCTGCCTGCACCAGGAG
EHNF4α	CAGGGTCTGAGCCCTATAAG	CGTCTTCCTTTGCCGTGACC
EHNF6	GAGCTGAGCACTGTCAGCAA	AGTTTGTGGTTCTTCCTTCA
EHNF1α	GTGTCCTCCAGCAGCCTGGT	GGCTCAGGTCCTCCAGGCAG
EHEX	AGTTTGGACAGTTCCTGTGA	CATGCCAATGCCAGTGGTCA
ECEBPα	AAAGGGGTGGAAACATAGGG	GGAGAGGCGTGGAACTAGAG

* The E denotes the endogenous.
