# Supplementary material for: Generation of hepatocyte- and endocrine pancreatic-like cells from human induced endodermal progenitor cells
Source: PLoS One. 2018 May 11;13(5):e0197046. doi: 10.1371/journal.pone.0197046 (PMC5947914; doi:10.1371/journal.pone.0197046)
Supplement: S5 Table — (PDF) [file pone.0197046.s020.pdf]

S5 Table. List of qRT-PCR primers used for Primitive endoderm, Mesendoderm, hepatocytes, pancreatic endocrine cells (Exon-exon spanning primers or CDS-3'UTR or 5'UTR-CDS).

Genes	Forward primer sequence	Reverse primer sequence
*PPIG (HKG)**	CTTGTCAATGGCCAACAGAGG	GCCCATCTAAATGAGGAGTTGGT
SOX7	GCCTGTGCAACAAGAGTGAA	GTACCCTGGGTCTTTGGTCA
GATA6	CCCTACTCGCCCTACGTG	GGACAGGTCCTCCAGCAG
GSC	TCTCAACCAGCTGCACTGTC	CCAGACCTCCACTTTCTCCTC
EOMES	AACAACACCCAGATGATAGTC	TCATAGTTGTCTCTGAAGCCT
FGF8	GAGACGGGCCTCTACATCTG	GTGTAGTTGTTCTCCAGCAC
CER1	GGACAGTGCCCTTCAGCCAGACTA	TGCCTGCCAAGTTCACCACGA
CXCR4	CACCGCATCTGGAGAACCA	GCCCATTTCCTCGGTGTAGTT
CKIT	TGGGCCACCGTTTGGAAAGC	GGGTGTGGGGATGGATTTGCTCTTT
E-CADHERIN	CGAACTATATTCTTCTGTGAGAGG	GATAGATTCTTGGGTTGGGTC
EPCAM	CTGGCCGTAAACTGCTTTGT	AGCCCATCATTGTTCTGGAG
OCCULUDIN	TCTGCAGGCACACAGGACGTG	AACCACTTCAGGAACCGGCGT
ALB	ATGCTGAGGCAAAGGATGTC	AGCAGCAGCACGACAGAGTA
AFP	TGAGCACTGTTGCAGAGGAG	GTGGTCAGTTTGCAGCATTC
AAT	AGGGCCTGAAGCTAGTGGAT	TCCTCGGTGTCCTTGACTTC
MRP2	CGATATACCAATCCAAGCCTC	GAATTGTCACCCTGTAAGAGTG
CYP3A4	TTCCTCCCTGAAAGATTCAGC	GTTGAAGAAGTCCTCCTAAGCT
TTR	AAACCAGTGAGTCTGGAGAG	CTGTGAATACCACCTCTGCA
G6PC	GTGTCCGTGATCGCAGACC	GACGAGGTTGAGCCAGTCTC
PROX1	TCACCTTATTCGGGAAGTGC	GGAGCTGGGATAACGGGTA
PTF1A	ACGACTTCTTCACCGACCAG	TGGTGGCTAAGGAACTCCAC
PDX1	TCCACCTTGGGACCTGTTTA	GTGTGTTAGGGAGCCTTCCA
NGN3	TCTCTATTCTTTTGCGCCGG	CTTGGACAGTGGGCGCAC
NKX6.1	CTTCCCGTCTTTGTCCAACAA	CCATCTTCTGGCCCGGAGTGA
NEUROD1	TAAGACGCAGAAGCTGTCCA	CTGCTCAGGCAGAAAAGTCC
NKX2.2	CAACACAAAGACGGGGTTTT	GTTGTCGCTGCTGTCGTAGA
ARX	ACAGCGTGTGCCTCTCTGC	TCGGGCCTCGGTCAAGTCC
PAX4	CAACCGAGTCCTGCGGGCAT	GCCAGCTTTCCACGGGCCAC
PAX6	CCCAAGAGCAAATTGAGGCCC	CTCTTCTCCATTTGGCCCTTCGA
MAFA	TCATCCGGCTCAAGCAGAAG	TCTCGCTCTCCAGAATGTGC
MAFB	GCCTGCGCTAATTGTAGGAG	CAAAAGCAGGGAAAGAAACG
INS	ATCAAGCACATCACTGTCCT	TGTAGAAGAAGCCTCGTTCC
GCG	GTTCCCTTCAAGACACAGAG	GGCAATGTTATTCCTGTTCC
SST	GAGGCTTGAGCTGCAGAGAT	TCGCTGAAGACTTGGAGGAT
AMYLASE	CAGGAGGTAATTGATCTGGGT	TTTGTACAGCCTAGCATCCC
HNF1B	TCACAGATACCAGCAGCATCAGT	GGGCATCCCAGGCTTGTA
KRT19	CGACTACAGCCACTACTACAC	GGTGGCACCAAGAATCTTGTC
LGR5	AAGAACACTGACTCTGAATGG	TAGCACTTGGAGATTAGGTAACTG
NKX2.5	ACCTCAACAGCTCCCTGACTCT	ATAATCGCCGCCACAAACTCTCC

* The HKG denotes the house keeping gene.
