# Supplementary material for: Generation of hepatocyte- and endocrine pancreatic-like cells from human induced endodermal progenitor cells
Source: PLoS One. 2018 May 11;13(5):e0197046. doi: 10.1371/journal.pone.0197046 (PMC5947914; doi:10.1371/journal.pone.0197046)
Supplement: S6 Table — (PDF) [file pone.0197046.s021.pdf]

S6 Table. List of primary and secondary antibodies used for immunostaining and immunohistochemistry

Antibody	Catalog number	Company	Fixation	Dilution	Blocking	Secondary Antibody
OCT4	sc-8628	Santa Cruz	4% PFA	1:200	5% Donkey serum	Donkey anti Rb-Alexa Fluor 488 (Invitrogen)
CXCR4-PE	12-9991	eBioscience	4% PFA	1:100	5% Donkey serum	-NA-
SOX17	AF1924	R&D	4% PFA	1:200	5% Donkey serum	Donkey anti Gt-Alexa Fluor 555 (Invitrogen)
KRT18	sc-32329	Santa Cruz	4% PFA	1:250	5% Donkey serum	Donkey anti Ms-Alexa Fluor 555 (Invitrogen)
ALB	Q 0328	Dako	4% PFA	1:4000	5% Donkey serum	Donkey anti Rb-AF 555 or 488 (Invitrogen)
AFP	A 0008	Dako	4% PFA	1:600	5% Donkey serum	Donkey anti Rb-AF 555 or 488 (Invitrogen)
AAT	A 0012	Dako	4% PFA	1:2000	5% Donkey serum	Donkey anti Rb-AF 555 or 488 (Invitrogen)
HNF4A	ab41898	Abcam	4% PFA	1:200	5% Donkey serum	Donkey anti Ms-Alexa Fluor 555 (Invitrogen)
PDX1	AF2419	R&D	4% PFA	1:200	5% Donkey serum	Donkey anti Gt-AF 555 or 488 (Invitrogen)
NGN3	MAB3444	R&D	4% PFA	1:100	5% Donkey serum	Donkey anti Ms-Alexa Fluor 555 (Invitrogen)
INS	A 0564	Dako	4% PFA	1:200	5% Goat serum	Goat anti GP-Alexa Fluor 555 (Invitrogen)
GCG	ab108426	Abcam	4% PFA	1:200	5% Donkey serum	Donkey anti Rb-Alexa Fluor 555 (Invitrogen)
SST	sc-7819	Santa Cruz	4% PFA	1:200	5% Donkey serum	Donkey anti Gt-Alexa Fluor 555 (Invitrogen)
CHGA (Neuroendocrine cell)	sc-13090	Santa Cruz	4% PFA	1:500	5% Donkey serum	Donkey anti Rb-Alexa Fluor 555 (Invitrogen)
Lysozyme (Paneth cells)	A0099	Dako	4% PFA	1:500	5% Donkey serum	Donkey anti Rb-Alexa Fluor 555 (Invitrogen)
Villin-C (Brush border microvilli)	SC-7672	Santa Cruz	4% PFA	1:50	5% Donkey serum	Donkey anti Gt-Alexa Fluor 488 (Invitrogen)
