# Supplementary material for: Generation of hepatocyte- and endocrine pancreatic-like cells from human induced endodermal progenitor cells
Source: PLoS One. 2018 May 11;13(5):e0197046. doi: 10.1371/journal.pone.0197046 (PMC5947914; doi:10.1371/journal.pone.0197046)
Supplement: S7 Table — (PDF) [file pone.0197046.s022.pdf]

S7 Table. List of primary and secondary antibodies used for immunostaining and immunohistochemistry

Antibody	Catalog number	Company	Fixation	Dilution	Blocking	Secondary Antibody
MUCIN2 (Goblet	sc-15334	Santa Cruz	4% PFA	1:500	5% Donkey serum	Donkey anti Rb-Alexa Fluor 555 (Invitrogen)
CDX2 (Intestine)	MU392A-UC	Biogenex	4% PFA	1:500	5% Donkey serum	Donkey anti Ms-Alexa Fluor 555 (Invitrogen)
C-peptide	ab1973	Abcam	4% PFA	1:250	5% Donkey serum	Donkey anti Ms-Alexa Fluor 555 (Invitrogen)
CK19	M0888	Dako	4% PFA	1:200	5% Donkey serum	Donkey anti Ms-Alexa Fluor 555 (Invitrogen)
NKX6.1	F55A10	DSHB	4% PFA	1:10	5% Donkey serum	Donkey anti Ms-Alexa Fluor 555 (Invitrogen)
TUBB3	AB9354	Millipore	4%PFA	1:1000	5% Donkey serum	Donkey anti Chi-Alexa Fluor 647 (Invitrogen)
NKX2.5	Sc-12514	Santa Cruz	4%PFA	1:100	5% Donkey serum	Donkey anti Gt-Alexa Fluor 555 (Invitrogen)

Abbreviations: Ms-mouse, Rb-rabbit, Gt-Goat,GP-guniea pig, Chi- Chicken.
