# Supplementary material for: Generation of hepatocyte- and endocrine pancreatic-like cells from human induced endodermal progenitor cells
Source: PLoS One. 2018 May 11;13(5):e0197046. doi: 10.1371/journal.pone.0197046 (PMC5947914; doi:10.1371/journal.pone.0197046)
Supplement: S8 Table — (PDF) [file pone.0197046.s023.pdf]

S8 Table. List of isotype antibodies used for immunostaining and immunohistochemistry

Isotypes antibodies	Company	Catalog no
Mouse IgG2a	Sigma	M9144
Mouse IgG1	Dako	X093101
Rabbit IgG	BD pharmingen	550875
Goat IgG	Jackson	JACK005-000-0020
Rabbit serum	Dako	X0902
PE-Rat IgG2bk	ebiosciences	12-4031
Guniea pig serum	Jackson	006-000-001
Chicken serum	Jackson	003-000-120
Hoechst	Sigma	33258
Prolong Gold with DAPI	Life technologies	P36931
