# Supplementary material for: Generation of hepatocyte- and endocrine pancreatic-like cells from human induced endodermal progenitor cells
Source: PLoS One. 2018 May 11;13(5):e0197046. doi: 10.1371/journal.pone.0197046 (PMC5947914; doi:10.1371/journal.pone.0197046)
Supplement: S9 Table — (PDF) [file pone.0197046.s024.pdf]

S9 Table. List of FACS antibodies

Antibodies	Company (Catalog no)	Label
CD34	BD Pharmingen (555821)	FITC
CD45	BD Pharmingen (555482)	FITC
CD44	BD Pharmingen (555478)	APC
CD73	BD Pharmingen (550257)	PE
CD117(CKIT)	eBioscience (17-1179)	APC
CXCR4 (CD184)	eBioscience (12-9999	PE
CD146	BD Pharmingen (550315)	PE
CD140a	BD Pharmingen (556002)	PE
CD140b	BD Pharmingen (558821)	PE
CD271	BD Pharmingen (557196)	PE
MHC Class II	BD Pharmingen (555812)	PE
MHC Class I	BD Pharmingen (555555)	APC
CD90	BD Pharmingen (559869)	FITC
ALP	R&D systems (FAB1448A)	APC
CD31	BD Pharmingen (555446)	PE
CD105	BD Pharmingen (FAB10971A)	APC
Mouse IgG1k isotype	BD Pharmingen (555748)	FITC
Mouse IgG2b isotype	BD Pharmingen (559532)	FITC
Mouse IgG1k isotype	BD Pharmingen (555749)	PE
Mouse IgG2a isotype	BD Pharmingen (555574)	PE
Mouse IgG1k isotype	BD Pharmingen (555751)	APC
